# Pro-Resolving FPR2 Agonists Regulate NADPH Oxidase-Dependent Phosphorylation of HSP27, OSR1, and MARCKS and Activation of the Respective Upstream Kinases

**DOI:** 10.3390/antiox10010134

**Published:** 2021-01-19

**Authors:** Rosario Ammendola, Melania Parisi, Gabriella Esposito, Fabio Cattaneo

**Affiliations:** Department of Molecular Medicine and Medical Biotechnology, School of Medicine, University of Naples Federico II, 80131 Naples, Italy; rosario.ammendola@unina.it (R.A.); melania.parisi@unina.it (M.P.); gabriella.esposito@unina.it (G.E.)

**Keywords:** formyl peptide receptors, NADPH oxidase (Nicotinamide Adenine Dinucleotide Phosphate oxidase), reactive oxygen species, annexin A1, HSP-27, OSR1 (Oxidative-Stress-Responsive kinase 1), MARCKS (Myristolated Alanine-Rich C-Kinase Substrate), inflammation

## Abstract

Background: Formyl peptide receptor 2 (FPR2) is involved in the pathogenesis of chronic inflammatory diseases, being activated either by pro-resolving or proinflammatory ligands. FPR2-associated signal transduction pathways result in phosphorylation of several proteins and in NADPH oxidase activation. We, herein, investigated molecular mechanisms underlying phosphorylation of heat shock protein 27 (HSP27), oxidative stress responsive kinase 1 (OSR1), and myristolated alanine-rich C-kinase substrate (MARCKS) elicited by the pro-resolving FPR2 agonists WKYMVm and annexin A1 (ANXA1). Methods: CaLu-6 cells or p22phox^Crispr/Cas9^ double nickase CaLu-6 cells were incubated for 5 min with WKYMVm or ANXA1, in the presence or absence of NADPH oxidase inhibitors. Phosphorylation at specific serine residues of HSP27, OSR1, and MARCKS, as well as the respective upstream kinases activated by FPR2 stimulation was analysed. Results: Blockade of NADPH oxidase functions prevents WKYMVm- and ANXA1-induced HSP-27(Ser82), OSR1(Ser339) and MARCKS(Ser170) phosphorylation. Moreover, NADPH oxidase inhibitors prevent WKYMVm- and ANXA1-dependent activation of p38MAPK, PI3K and PKCδ, the kinases upstream to HSP-27, OSR1 and MARCKS, respectively. The same results were obtained in p22phox^Crispr/Cas9^ cells. Conclusions: FPR2 shows an immunomodulatory role by regulating proinflammatory and anti-inflammatory activities and NADPH oxidase is a key regulator of inflammatory pathways. The activation of NADPH oxidase-dependent pro-resolving downstream signals suggests that FPR2 signalling and NADPH oxidase could represent novel targets for inflammation therapeutic intervention.

## 1. Introduction

G protein coupled receptors (GPCRs) constitute the largest family of cell surface receptors in humans with more than 800 genes [1]. Such a significant number of GPCRs ensures cellular responses to several stimuli including signalling peptides, hormones, and neurotransmitters, and thus the control of various biological functions [2]. GPCRs transduce intracellular signals through the activation of heterotrimeric G proteins [3] with the resultant generation of second messengers that regulate the activity of several enzymes and the control of cellular functions [4]. Several studies have shown that reactive oxygen species (ROS) generation increases in response to GPCR stimulation, and that low levels of ROS act as signalling molecules playing a key role in several cellular functions. In contrast, higher amounts of ROS generated during heart failure, diabetes, hypertension, and ischemia-reperfusion injury contribute to apoptosis and cell death.

Several cytosolic enzymes, such as xanthine oxidoreductase, cyclo- and lipoxygenases, peroxisomal oxidases, and members of the cytochrome P450 family produce ROS, even though this is not their specific role. ROS can also be generated at the mitochondrial level as by-products of electron transport chain [5]. The family of NADPH oxidases represents the only group of enzymes whose unique function is ROS generation. Furthermore, since they produce ROS also in response to activated GPCRs or tyrosine kinase receptors (TKRs), this family of oxidases shows the peculiar property to link extracellular stimuli to intracellular signalling pathways [6].

The phagocyte NADPH oxidase is composed of the membrane-bound subunits gp91^phox^ and p22^phox^, both forming the cytochrome b558, and the cytosolic subunits p67^phox^, p47^phox^, p40^phox^, and GTPase Rac. These latter components are localized in the cytosol under unstimulated conditions, thus, preventing the undesirable production of superoxide anion that can be harmful for the cells. Phosphorylation and membrane translocation of the cytosolic subunits is required for their association with the cytochrome b558 and, in turn, for NADPH oxidase activation [6]. Subunit p67^phox^ acts as the activator of gp91^phox^, whereas p47^phox^ acts as the organizer ensuring that all subunits are properly aligned for optimal function. Subunit p40^phox^ positively or negatively regulates NADPH oxidase function, and the small monomeric GTPases Rac1and Rac2 are required for the full activation of the enzymatic complex [7,8,9,10]. Subunits gp91^phox^ and p22^phox^, as well as the cytosolic subunits of the phagocyte oxidase are also expressed in nonphagocytic cells [11,12,13,14]. Interestingly, in neuroblastoma, endothelial, epithelial, fibroblasts, and lung cancer cells, we previously identified functional NADPH oxidases that generated low amounts of ROS involved in intracellular signalling cascades [15,16,17,18,19,20].

In nonphagocytic cells, binding of several ligands to their cognate GPCRs, such as angiotensin II [21], urotensin-II [22], endothelin-1 [23], thrombin [24], extracellular ATP [25], and adenosine [26] promotes NADPH oxidase-dependent ROS generation. In neutrophils, the first evidence that GPCRs can activate NADPH oxidase came from the demonstration that the binding of N-formylmethionyl-leucyl-phenilalanine (N-fMLP) to formyl peptide receptor 1 (FPR1), a member of the GPCR family, resulted in the ERK- and PKC-dependent phosphorylation of NADPH oxidase cytosolic subunits, which translocated on membrane and formed an enzymatic active complex [27,28]. 

In humans, three different forms of formyl peptide receptor 2 (FPR) (FPR1, FPR2, and FPR3) are expressed. Among them, FPR2 is the more ubiquitously receptor, mainly expressed in cells of the bone marrow, immune system, gastro-intestinal tract, female organ tissues, endocrine glands, brain, liver, gallbladder, and pancreas [29]. FPRs are responsible for neutrophil chemotaxis, but FPR1 and FPR2 also play pivotal roles in the progression of multiple diseases. For example, FPR2 promotes the malignancy of colon cancer and FPR1 is involved in the progression of glioblastoma [30,31]. Most FPR2 ligands, besides inducing chemotaxis, calcium flux, and phagocytosis, also stimulate many other cell functions [32], such as proinflammatory processes and pro-resolving or anti-inflammatory pathways. This duality in FPR2 is determined by the nature of the ligands. Bacterial and mitochondrial formylated peptides activate a proinflammatory cell response, while annexin A1 (ANXAA1) and lipoxin A4 (LXA4) are some of the better-known anti-inflammatory FPR2 ligands [33]. The ability of FPR2 to convey opposite signals depends on different receptor domains used by pro-resolving or proinflammatory agonists [34], and the switch between FPR2-mediated proinflammatory and anti-inflammatory cell responses is due to conformational changes of the receptor upon ligand binding [35]. On the one hand, pro-resolving mediators, such as ANXA1 and LXA4, in contrast to anti-inflammatory molecules, reduce inflammation without compromising the host defenses against pathogens [36,37]. On the other hand, serum-amyloid alpha (SAA) acts as a proinflammatory ligand on FPR2 by activating the NF-κB pathway and by inducing the expression of proinflammatory factors [38].

Although several FPR ligands are small molecules or non-peptides, the majority of agonists are small peptides with origins ranging from host and multicellular organisms to viruses and bacteria. The presence of formylated methionine in the peptide suggests generally an activator of FPR1, while FPR2 is less dependent upon this residue [39]. Mammalian cell mitochondria, well-known for being bacterial in origin, contain formylated peptides that elicit mainly FPR1-mediated responses. Nevertheless, while bacterial formylated peptides are considered to be pathogen-associated molecular patterns (PAMPs), the mitochondrial peptides are associated with cellular damage, and thus are considered to be damage-associated molecular patterns (DAMPs) that trigger inflammatory responses [40]. Furthermore, FPR1 favors binding of short and flexible structures while FPR2 has binding preference for longer amphipathic peptides which contain alpha helices [41]. Therefore, even though the majority of formylated microbial peptides preferentially activate FPR1, the favored receptor for non-formylated peptides is FPR2 [39]. Many of these non-formylated microbe-derived peptides are viral, such as those derived from the human immunodeficiency virus envelope proteins [42,43], hepatitis C virus, HKU-1 coronavirus, and herpes simplex virus [44,45,46]. There are few non-viral and non-formylated FPR2 microbe-derived agonists, such as some *Enterococcus faecium* strains [47] and agonists belonging to PAMPs, such as those derived from *Helicobacter pylori* [48]. 

Many different host-derived ligands elicit different biological implications. Among these amyloid β-42 (Aβ-42), a peptide fragment well-documented in Alzheimer’s disease, the prion peptide fragment PrP106–126, the neuropeptide pituitary adenylate cyclase-activating polypeptide 27, and the vasoactive intestinal peptide efficiently interact with FPR2 [49]. Other host-derived FPR2 ligands include the SHAAGtide sequence, various domains from the urokinase-type 1 plasminogen activator receptor, the F2L peptide derived from the N-terminus of the heme-binding protein, and the chemokine-like peptide derived from family with sequence similarity 3 (member D) [29].

In addition to endogenous peptides, many ligands secreted by cells in response to tissue damage bind FPR1 and FPR2. For instance, ANXA1 activates both FPR1 and FPR2 [50,51], but peptides derivatives of ANXA1, including Ac1-25, Ac2-26, and Ac9-25 activate only FPR2 [52,53]. Furthermore, SAA is an endogenous FPR2 agonist secreted by liver or macrophages in response to inflammatory stress and, more notably, tissue damage [54]. Finally, the human cathelicidin LL-37 is an antimicrobial peptide that induces B cell activation and proliferation via FPR2 [18]. 

Therefore, FPR1 binds efficiently N-fMLP and other formylated peptides, whereas FPR2 is a promiscuous receptor activated by the synthetic peptide WKYMVm [55], LXA4, ANXA1 [56], SAA, and by peptides or lipids of different origin. FPR3 shares some non-formylated peptide ligands with FPR2 [57].

FPR2 is highly expressed in immune system cells, but it is also functionally expressed onto cellular membrane of several nonphagocytic cells [19,58] and onto nuclear membranes of CaLu-6 and AGS cells, where it elicits intranuclear signalling pathways implicated in gene expression regulation [59]. FPR2 stimulation triggers the activation/phosphorylation of several signalling proteins, such as phospholipase A2 (PLA2), phospholipase C (PLC), phospholipase D (PLD), phosphatidylinositol-3-kinase (PI3-K), protein kinase B (PKB or Akt), protein kinase C (PKC), p38MAPK, extracellular response kinases 1 and 2 (ERK1/2), and c-Src [19,60,61,62,63], which modulate proliferation, cell growth, migration, intracellular communication, apoptosis, survival, differentiation, and other critical intracellular functions [64,65]. Phosphorylation of p47^phox^ and p67^phox^ and, in turn, NADPH oxidase activation represents one of the targets of the FPR2-induced signalling cascade [18,20]. 

A characteristic of the inflammation process is leukocyte infiltration at disease sites in response to PAMPs or DAMPs which interact with chemotactic GPCRs, such as the members of the FPR family. These assist the organism in counteracting bacterial infections by facilitating the trafficking of phagocytes to the site of bacterial invasion [66] and triggering intracellular signalling cascades that modulate the survival and the phagocytic activity of infiltrating cells [67]. The observation that the deletion of the Fpr1 gene reduced inflammation in an experimental mouse model of endometriosis [68] and that FPRs were mainly expressed on neutrophils and macrophages, suggests that these receptors predominantly govern a proinflammatory response which results in chemotaxis, degranulation, and oxidative burst during infection. The release of endogenous FPR ligands can influence severe diseases associated with inflammation, including systemic inflammatory response syndrome [69], Alzheimer′s disease, amyloidosis, prion disease, obesity, diabetes [70], and several types of cancer [71,72,73,74]. However, FPRs can also promote the resolution of inflammation. In fact, during an acute inflammatory response, leukocytes migrate towards an increasing concentration gradient of chemotactic factors but, in a later inflammatory stage, a number of anti-inflammatory FPR2 ligands are generated. These could influence the leukocyte migratory response [75,76]. Thus, the production of endogenous anti-inflammatory mediators increases at a later phase of the innate immune response to induce resolution of the inflammation [76]. The pro-resolving signalling in some cell types is mainly attributed to the findings with endogenous FPR1/FPR2 ligands, ANXA1 and its peptide fragments. The molecular basis for the capacity of these agonists to trigger a divergent signalling pathway is attributed to their binding to different domains of the receptors [77].

Phosphorylation/dephosphorylation is the most common covalent modification of proteins. It is mainly regulated by protein phosphatases through the reversible oxidative inhibition of reactive cysteine residues [78,79,80,81]. This molecular mechanism highlights the key role of ROS in the regulation of phosphorylation/dephosphorylation of several proteins, as well as in TKRs transactivation [82]. We previously demonstrated that FPR stimulation induces NADPH oxidase-dependent trans-phosphorylation of several TKRs, such as epidermal growth factor receptor (EGFR), hepatocyte growth factor receptor (c-Met), vascular endothelial growth factor receptor 2 (VEGFR2), and neurotrophin receptor TrkA, and that phospho-tyrosine residues of these receptors provide docking sites for recruitment, phosphorylation, and triggering of signalling proteins [15,16,17,20].

By using a phospho-proteomic analysis we demonstrated that FPR2 stimulation by WKYMVm or ANXA1 induces the phosphorylation of several proteins involved in metabolic processes and biological regulation [56,83], including heat shock protein 27 (HSP27), oxidative stress responsive kinase 1 (OSR1), and myristolated alanine-rich C-kinase substrate (MARCK). Herein, we analyse the mechanism linking pro-resolving agonist stimulation of FPR2 with specific phosphorylation of HSP27(Ser82), OSR1(Ser339), MARCK(Ser170), and their respective upstream kinases.

## 2. Materials and Methods

### 2.1. Reagents

CaLu-6 cell line (ATCC, Manassas, VA, USA) and p22phox^Crispr/Cas9^ CaLu-6 cells were cultured in Dulbecco’s modified Eagle’s medium (DMEM) (Thermo Fisher Scientific, Monza, Italy) supplemented with 10% foetal bovine serum (FBS) (Invitrogen Corp., Carlsbad, CA, USA). Cells were grown until they reached 80% confluence, serum-deprived for 24 h, and then stimulated for 5 min with the peptide WKYMVm (Primm, Milan, Italy) at the final concentration of 10 μM, or with ANXA1 (Abcam Inc., Cambridge, MA, USA) at the final concentration of 10 nM. Cells were also preincubated with apocynin (Sigma Chemical, St. Louis, MO, USA) for 2 h at a final concentration of 5 mM, or with *N*-acety-l-cysteine (NAC) (Sigma Chemical, St. Louis, MO, USA) for 90 min at a final concentration of 100 μM, before FPR2 stimulation. 

Anti-phospho-HSP-27 (S82), anti-phospho-p38 MAPK (T180/Y182), and anti-phospho-PI3K (Y458) were from Cell Signalling Technology (Denvers, MA, USA). Anti-phospho-OSR1 (S339) was purchased from Signalway Antibody (Baltimore, MD, USA) and anti-phospho-MARCKS (S170) was from GeneTex (Irvine, CA, USA). Antitubulin, anti-phospho-PKCδ (T507), anti-GAPDH, anti-rabbit, anti-mouse, and anti-goat antibodies were purchased from Santa Cruz Biotechnology (Irvine, CA, USA). Protein A, i.e., horseradish peroxidase was from Thermo Scientific (Little Chalfont, Buckinghamshire, UK).

### 2.2. The p22phox ^Crispr/Cas9^ Double-Nickase CaLu-6 Cells

The p22phox^Crispr/Cas9^ cells were obtained by transfecting CaLu-6 cells with Double Nickase Plasmid (Santa Cruz Biotechnology, Irvine, CA, USA), according to manufacturer’s instructions. Briefly, 1.5 × 105 CaLu-6 wild type cells were seeded in DMEM containing 10% FBS without antibiotics in a single well of a multi-6 multi-well tissue culture plate for 24 h. Then, cells were transfected with 3 μg of p22phox Double Nickase Plasmid DNA or 3 μg of control double nickase plasmid, as negative control, by using 10 μL of UltraCruz^®^ transfection Reagent, according to the supplier’s instructions. Double Nickase Plasmid consists of a pool of plasmid pairs each coding for the mutated Cas9 nuclease D10A, a target-specific guide RNA of 20 nt. One plasmid in the pair contains a puromycin resistance gene for selection; the other plasmid in the pair contains a green fluorescence protein (GFP) marker to visually confirm transfection. Culture medium was replaced with a selective medium containing 1 µg/mL puromycin (Santa Cruz Biotechnology, Dallas, TX, USA) for 48 h. Transfected cells were selected in puromycin medium for 5 days and selective medium was replaced every 2 days. Single cell clones were isolated, cultured separately, and tested by Western blotting to analyse p22phox expression (Supplementary Figure S1). Colonies knockout for p22phox gene were isolated in order to obtain p22phox^Crispr/Cas9^ pool cells (p22phox^Crispr/Cas9^), whereas control double nickase plasmid-transfected cells, expressing p22phox, were collected in order to obtain CaLu-6-control^Crispr/Cas9^ cells (CTR). 

### 2.3. Protein Extraction and Western Blot

Whole lysates were obtained from 24-h serum-starved CaLu-6 cells stimulated or not for 5 min with 10 μM WKYMVm or 10 nM ANXA1 in the presence or absence of the appropriate amounts of NADPH oxidase specific inhibitors, as described elsewhere [56]. Whole lysates were also obtained from p22phox^Crispr/Cas9^ and CTR cells. Briefly, cells were rinsed in cold phosphate buffered saline (PBS) and lysed by incubation with RIPA buffer (50 mM Tris-HCl, pH 7.4, 150 mM NaCl, 1% NP-40, 1 mM EDTA, 0.25% sodium deoxycholate, 1 mM NaF, 10 μM Na_3_VO_4_, 1 mM phenylmethylsulfonylfluoride, 10 μg/mL aprotinin, 10 μg/mL pepstatin, and 10 μg/mL leupeptin) for 45 min at 4 °C [84]. Proteins concentration was measured with a Bio-Rad protein assay (BioRAD, Hercules, CA, USA). Equal amounts (sixty micrograms) of lysates were resolved on 10% SDS-PAGE and transferred onto PVDF membranes. Reagents for SDS-PAGE were provided by Bio-Rad (Hercules, CA, USA). Membranes were probed with phospho-specific primary antibodies followed by incubation with the appropriate horseradish peroxidase-conjugated secondary antibodies. reactive bands were detected by ChemiDoc XRS Image System (Bio-Rad Laboratories, Hercules, CA, USA) and quantified by using Image Lab software (Bio-Rad, Hercules, CA) [85]. The same filters were reprobed with an antitubulin antibody, as a control for protein loading. 

### 2.4. Statistical Analysis

Statistical analyses were evaluated by unpaired t-test to compare the mean of two independent groups of experiments or by one-way analysis of variance (ANOVA); and GraphPad Prism 7 (GraphPad Software Inc., San Diego, CA, USA) was used to compare more than two experiments. All data reported are representative of at least three or more independent experiments and are expressed as means ± standard error mean (SEM). A *p* value of less than 0.05 was considered to be statistically significant.

## 3. Results and Discussion

### 3.1. WKYMVm-Induced Heat Shock Protein 27 (HSP-27)(S82), Oxidative Stress Responsive Kinase 1 (OSR1)(S339), and Myristolated Alanine-Rich C-Kinase Substrate (MARCKS)(S170) Phosphorylation Depends on NADPH Oxidase Activity

Previously, we applied a phospho-proteomics approach to analyse phosphorylated proteins in WKYMVm-stimulated CaLu-6 cells and identified 290 phosphoproteins and 53 unique phospho-peptides mapping on 40 proteins [56]. We also demonstrated that the selective phosphorylation on Ser82 of HSP-27, Ser339 of OSR1, and Ser170 of MARCKS uniquely depends on FPR2 stimulation by WKYMVm.

**Figure 1:**
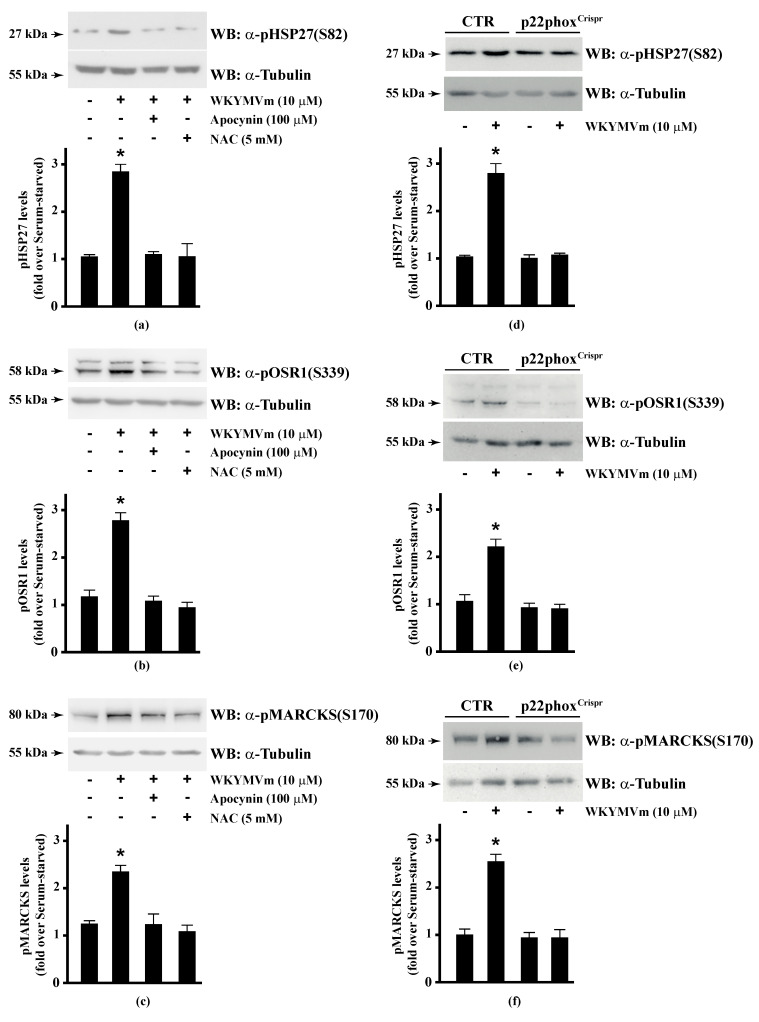
NADPH oxidase blockade functions prevents WKYMVm-induced heat shock protein 27 (*HSP27*)*,* oxidative stress responsive kinase 1 (*OSR1*) *and* myristolated alanine-rich C-kinase substrate (*MARCKs*) *phosphorylation.* (**a**–**c**) CaLu-6 cells were serum deprived for 24 h and stimulated with WKYMVm for 5 min or preincubated with 100 μM Apocynin or 5 mM *N*-acety-l-cysteine (NAC) before stimulation; (**d**–**f**) CaLu-6-control^Crispr/Cas9^ cells (CTR) and p22phox^Crispr/Cas9^ (p22phox^Crispr^) cells were serum starved for 24 h, and then stimulated with a scrambled hexapeptide or with 10 μM WKYMVm for 5 min. Sixty micrograms of whole lysates were resolved on 10% SDS-PAGE, transferred onto PVDF membrane and immunoblotted with (**a**,**d**) anti-phospho-HSP27(Ser82) (α-pHSP27(S82)), or (**b**,**e**) anti-phospho-OSR1(Ser339) (α-pOSR1(S339)), or (**c**,**f**) anti-phospho-MARCKS(Ser170) (α-pMARCKS(S170)) antibodies. An antitubulin (α tubulin) antibody was used as a control for protein loading. Bar graphs show the densitometric analysis performed on phosphorylated bands. Data are representative of at least three independent experiments. * *p* < 0.05 as compared with unstimulated cells.

Several FPR2 agonists trigger the phosphorylation of various intracellular signalling proteins and, in turn, NADPH oxidase-dependent ROS generation [15,16,17,18,20]. Therefore, we first wondered whether HSP-27(S82), OSR1(S339), and MARCKS(S170) phosphorylation depends on NADPH oxidase activation. To this aim, we preincubated CaLu-6 cells with apocynin, which prevents both p47^phox^ translocation and its interaction with p22^phox^ [86,87], or N-acetyl-l-cysteine (NAC), which plays a protective role against ROS [88], and then stimulated them with WKYMVm. In both cases, we observed a significant decrease in the WKYMVm-induced HSP-27(S82), OSR1(S339), and MARCKS(S170) phosphorylation (Figure 1, Panels a–c). By CRISPR/Cas9-based genome editing, we obtained a Calu-6 cell line expressing a not functional form of p22^phox^ (p22phox^Crispr/Cas9^). Significantly, stimulation of these cells with WKYMVm failed to induce HSP-27(S82), OSR1(S339), and MARCKS(S170) phosphorylation (Figure 1, Panels d–f).

HSP-27 is expressed in many cell types and tissues and its expression is increased by H_2_O_2_ [89]. It plays a key role in the regulation of protein degradation through the ubiquitin-proteasomal system [90], conferring cellular tolerance to heat and oxidative stress [91]. Phosphorylation of HSP-27 at Ser15, Ser78, and Ser82 residues [92] is crucial for the regulation of several cellular processes, such as prevention of apoptosis [93], resistance against oxidative stress [94], modulation of estrogen signalling [95], and actin polymerization [96]. Several stimuli, including oxidative stress, infection, ischaemia, cytokines, growth factors, and inflammatio alter the HSP-27 phosphorylation state. HSP-27 phosphorylation promotes its disassociation from multimers into monomers and dimers with several distinct functions [97]. In fact, phosphorylated HSP-27 exists as monomers or small oligomers and HSP-27 phosphorylation prevents its oligomerization into large multimers [98]. In its unphosphorylated monomeric form, HSP-27 inhibits actin polymerization [99], suggesting that phosphor-HSP-27 exerts its protective effect by reorganizing the cytoskeleton. On the one hand, phosphorylated HSP-27(Ser82) is present in macrophages in atherosclerotic lesions, indicating that this serine residue might be involved in the oxidative defense mechanisms [100,101] or in cell migration in atherosclerotic plaque. On the other hand, a selective depletion of HSP-27, phosphorylated at Ser15 [102], Ser78, and Ser82 residues has been observed in blood vessel walls of patients with ischemic heart disease [103]. These results are consistent with the hypothesis that phosphor-HSP-27 protects against vascular disease by stabilizing the actin cytoskeleton. Since many protective roles of HSP-27 are exerted by its phosphorylated form, cells showing a lower ratio of phosphor-HSP-27(Ser82) to total HSP-27 might be more vulnerable to oxidative stress and inflammation. Stimulation of some GPCRs or TKRs, such as β2-adrenoceptor or PDGFR, induces NADPH oxidase-dependent HSP-27 phosphorylation at not specified serine residues [104,105]. The observation that WKYMVm-induced HSP-27(Ser82) phosphorylation depends on NADPH oxidase activity strongly suggests that pro-resolving agonists trigger a protective redox response against inflammation and/or oxidative stress.

The WNK-SPAK/OSR1 kinase complex is composed of the kinases WNK and SPAK or the SPAK homolog OSR1 (oxidative stress-responsive kinase 1), which is involved in the modulation of kidney functions, blood pressure, cellular ion homeostasis, and hearing. WNK stimulates the kinase activity of OSR1 by phosphorylating conserved Thr185 and Thr243 residues within the catalytic T-loop kinase motif. OSR1 is also phosphorylated at Ser325, Ser339, and Ser373 residues in the S domain [106], but the role these phosphorylations is unclear, although some evidence suggests that Ser339 phosphorylation is involved in the binding of OSR1 to the scaffolding protein MO25, enhancing OSR1 catalytic activities [107]. However, although only phosphorylation of the T-loop is required to activate OSR1 [108], its kinase activity is increased upon Ser339 phosphorylation by mammalian target of rapamycin complex 2 (mTORC2) [109]. 

In phagocytes, WNK-OSR1-solute carrier (SLC) protein pathway represents a reversible switch controlling the anti- versus proinflammatory response. In SLC protein-deficient phagocytes, the canonical anti-inflammatory program is replaced by proinflammatory and oxidative stress-associated programs. This switch is due to disruption of the chloride-sensing pathway and of the chloride-sensing upstream kinases WNK1-SPAK/OSR1 [110]. Interestingly, Bartter’s syndrome and Gitelman’s syndrome are caused by mutations in the SLC protein family, as well as in WNK1-SPAK/OSR1 kinases that function upstream of SLC. In these syndromes, several inflammatory diseases arise at later stages [110], suggesting that the integrity and the activation of WNK1-SPAK/OSR-SLC pathway represent an anti-inflammatory response. Although it seems that ROS play a role in OSR1 phosphorylation [111], the involvement of NADPH oxidase-dependent ROS generation has never been proven. Our experiments show that WKYMVm triggers NADPH oxidase-dependent phosphorylation of OSR1 at Ser339 residue, which increases OSR1 activity [109] and, likely, its anti-inflammatory responses.

The membrane-binding protein MARCKS efficiently binds calmodulin (CaM) and is the main substrate of PKC in different cell types. It is involved in the regulation of cellular events requiring dynamic actin reorganization, such as cytoskeletal control, adhesion, and cell migration [112]. In resting cells, non-phosphorylated MARCKS is localized to the inner leaflet of the plasma membrane, where it stabilizes the cytoskeleton through actin cross-linking. Upon PKC phosphorylation, MARCKS translocates to the cytosol and actin cross-linking is reduced, thereby, efficiently relaxing the cellular cytoskeleton [113]. Specific phosphatases dephosphorylate MARCKS in the cytosol, restoring MARCKS ability to translocate to plasma membrane and to cross-link actin. In FPR1-stimulated neutrophils [114] and FPR2-stimulated IMR90 human fibroblasts [115], PKCδ translocates from cytosol to membrane. Because MARCKS phosphorylation occurs at the plasma membrane, PKCδ translocation is consistent with MARCKS phosphorylation and, in turn, with the increase of its detachment from the membrane and with the reduction in MARCKS affinity for CaM. Our present results demonstrate that blockade of NADPH oxidase functions prevents FPR2-induced MARCKS(Ser170) phosphorylation. The full activity of p22^phox^ is also required for MARCKS phosphorylation at Ser152 and Ser156 regulatory residues [116]. However, while MARCKS phosphorylation at Ser159 and Ser163 is mainly involved in inflammatory diseases [117,118], phosphorylation at Ser152, Ser156, and 170 residues seems to be involved in the regulation of cytoskeletal reorganization [116].

### 3.2. HSP-27(S82), OSR1(S339), and MARCKS(S170) Phosphorylation by Annexin A1 (ANXA1) Is Regulated by NADPH Oxidase

WKYMVm shows immunomodulatory and anti-inflammatory effects [119]. In fact, it enhances the levels of anti-inflammatory cytokines (IL-10 and TGFβ) and inhibits the generation of proinflammatory cytokines (TNF-α, IL-1β, and IL-6) [120]. Nevertheless, FPR2 can communicate both proinflammatory and anti-inflammatory signals and this ability is determined by the nature of the agonists that activate different signalling pathways, and by the formation of higher-order structures. The switch between proinflammatory and anti-inflammatory responses is caused by FPR2 conformational changes due to the agonists’ binding and to the ability of FPR2 to homodimerize or to heterodimerize with FPR1 or FPR3 [35]. 

On the one hand, ANXA1 transduces its pro-resolving effects through binding to FPR2 [35,121] and by promoting the formation of FPR2 homodimers or FPR1/FPR2 heterodimers and, in turn, the release of IL-10, TGFβ, and other anti-inflammatory cytokines. On the other hand, proinflammatory agonists, such as SAA, do not cause receptor homodimerization [35] and, in the absence of specialized pro-resolving mediators, signalling through FPR2 may be predominantly proinflammatory [122].

Previously, we demonstrated a potential FPR2 homodimerization upon WKYMVm stimulation, which indicates the triggering of anti-inflammatory signals; moreover, we showed that stimulation of CaLu-6 cells with ANXA1 induces HSP-27(S82), OSR1(S339), and MARCKS(S170) phosphorylation [56].

NADPH oxidase is as critical modulators of inflammation in neutrophils and macrophages and can also modulate acute inflammation and injury by regulation of transcriptional factors [123]. Thus, we analysed the involvement of NADPH oxidase in the regulation of HSP-27(S82), OSR1(S339), and MARCKS(S170) phosphorylation triggered by the pro-resolving ligand ANXA1. 

**Figure 2:**
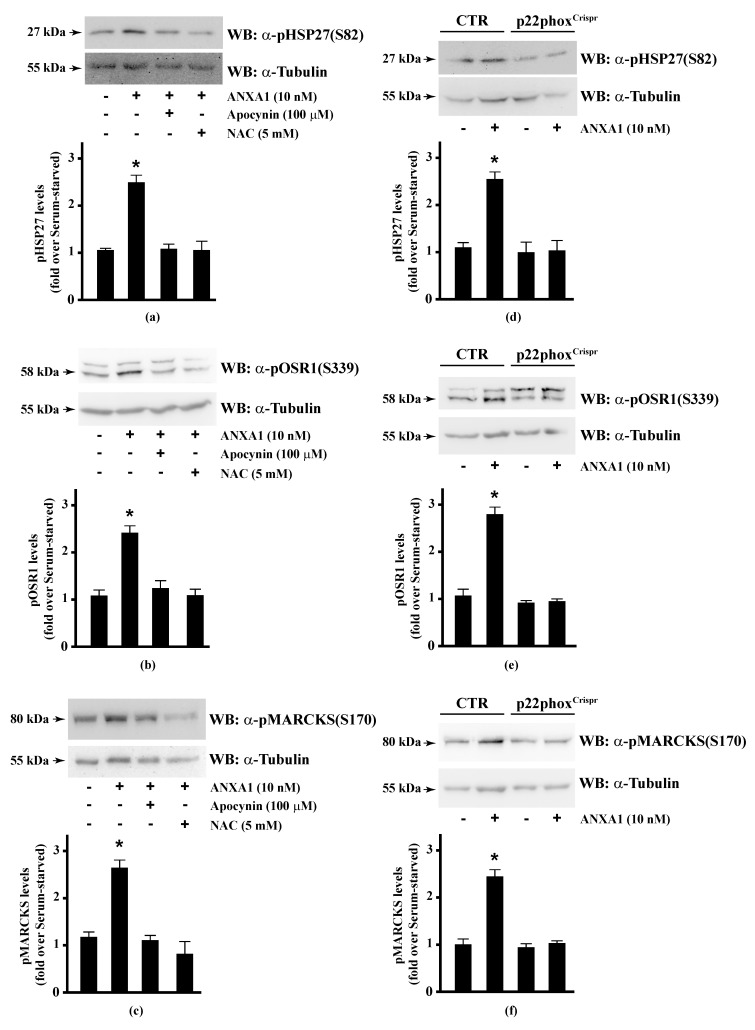
Annexin A1 (ANXA1)-induced HSP27, OSR1, and MARCKs phosphorylation depends on NADPH oxidase activity. (**a**–**c**) Serum-starved CaLu-6 cells were stimulated with 10 nM ANXA1 for 5 min in the presence or absence of the appropriate amounts of NADPH oxidase inhibitors; (**d**–**f**) CaLu-6-control^Crispr/Cas9^ cells (CTR) and p22phox^Crispr/Cas9^ (p22phox^Crispr^) cells were grown until they reached 80% of confluence, serum deprived for 24 h, and then stimulated with the vehicle or with 10 nM ANXA1 for 5 min. Total lysates (60 μg) were resolved on 10% SDS-PAGE and membranes were incubated with (**a**,**d**) anti-phospho-HSP27(Ser82) (α-pHSP27(S82)), or (**b**,**e**) anti-phospho-OSR1(Ser339) (α-pOSR1(S339)), or (**c**,**f**) anti-phospho-MARCKS(Ser170) (α-pMARCKS(S170)) antibodies. An antitubulin (α-tubulin) antibody was used as a control for protein loading. The data are representative of at least three independent experiments. Densitometric analysis was performed as described in Materials and Methods. * *p* < 0.05 as compared with unstimulated cells.

Therefore, we preincubated CaLu-6 cells with apocynin or NAC, and then stimulated them with ANXA1. Western blot analysis showed that both apocynin and NAC pretreatment significantly prevented ANXA1-induced HSP-27(S82), OSR1(S339), and MARCKS(S170) phosphorylation (Figure 2, Panels a–c). Consistently, these serine residues were not phosphorylated in p22phox^Crispr/Cas9^ Calu-6 cells stimulated with ANXA1 (Figure 2, Panels d–f). It should be noted that cathelicidin LL-37 and SAA, two FPR2 proinflammatory agonists [124,125], are unable to phosphorylate HSP-27 [35]. These results strongly suggest that some of the anti-inflammatory cellular responses triggered by the pro-resolving ligands WKYMVm or ANXA1 are regulated by NADPH oxidase activity.

### 3.3. Blockade of NADPH Oxidase Functions Prevents the Activation of the Kinases Upstream to HSP-27, OSR1, and MARCKS

Phosphorylation represents one of several types of reversible covalent post-translational modifications of many proteins. In phospho-proteins only a small subgroup of phosphorylated sites is subjected to regulation and single phospho-sites can be differently regulated, suggesting that proteins act as platforms that are able to integrate several incoming stimuli. Such integration can operate in an independent manner, in which each site can be phosphorylated independently from the others, or a dependent-manner, in which a first phosphorylation of a phospho-site is required for the subsequent phosphorylation events. Specific phospho-sites could expand the repertoire of molecular mechanisms of regulation or could be required for fine-tuning of switch properties that control different cellular functions [83,126,127].

**Figure 3:**
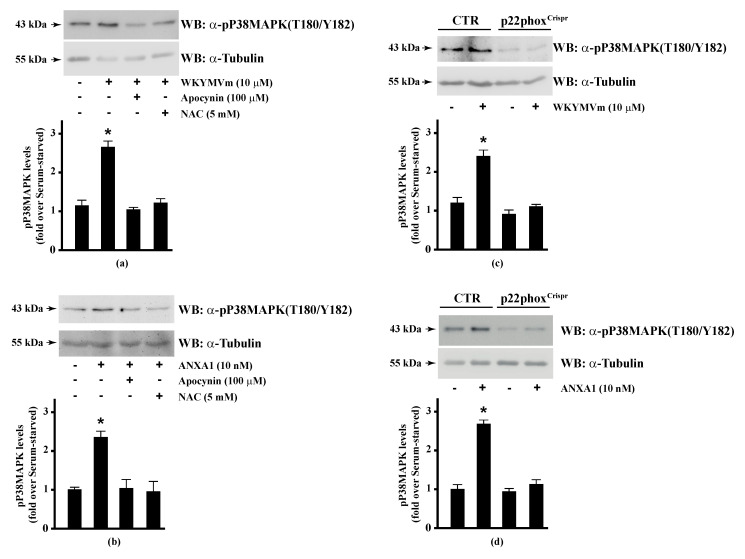
WKYMVm- and ANXA1-induced p38MAPK activation depends on NADPH oxidase-induced reactive oxygen species (ROS) generation. Serum-deprived CaLu-6 cells were stimulated with (**a**) 10 μM WKYMVm or (**b**) 10 nM ANXA1 for 5 min or preincubated with 100 μM apocynin or 5 mM NAC before stimulation. The CaLu-6-control^Crispr/Cas9^ cells (CTR) and p22phox^Crispr/Cas9^ (p22phox^Crispr^) cells were serum starved for 24 h, and then stimulated with (**c**) a scrambled hexapeptide or 10 μM WKYMVm for 5 min, or (**d**) the vehicle or 10 nM ANXA1 for 5 min. Whole lysates (60 μg) were resolved on 10% SDS-PAGE, transferred onto PVDF membrane, and incubated with an anti-phospho-p38MAPK (T180/Y182) antibody (α-p38MAPK). The data are representative of at least three independent experiments. Bar graphs show the densitometric analysis performed on phosphorylated bands. * *p* < 0.05 as compared with unstimulated cells. An antitubulin (α-tubulin) antibody was used as a control for protein loading.

Since HSP-27(S82), OSR1(S339), and MARCKS(S170) phosphorylation is regulated by NADPH oxidase activity, we analysed the regulation of the corresponding upstream kinases by ROS. HSP-27 is phosphorylated on Ser15, Ser78, and Ser82 residues by MAPKAP kinase 2/3/5 via activation of the p38MAPK pathway [92]. Western blot experiments showed that incubation of Calu-6 cells with WKYMVm or ANXA1 significantly induced p38MAPK phosphorylation, which was prevented by preincubation with apocynin or NAC (Figure 3, Panels a, b), suggesting that p38MAPK phosphorylation requires NADPH oxidase activity. Accordingly, both FPR2 agonists do not induce p38MAPK phosphorylation in p22phox^Crispr/Cas9^ cells (Figure 3, Panels c, d). p38MAPK phosphorylation in WKYMVm- and ANXA1-stimulated cells has been observed in several experimental systems [128,129,130,131].

The proinflammatory FPR2 ligand SAA increases NADPH oxidase-dependent ROS generation which activates the p38MAPK/NF-κB pathway and, in turn, promotes the release of inflammatory factors (IL-1β, IL-6, IL-8, IL-17, TNF-α and MCP-1) [132]. In addition, ANXA1 induces a p38MAPK-HSP-27 signalling signature leading to production of the anti-inflammatory cytokine IL-10 both in vitro and in vivo [35]. These observations suggest that p38MAPK is activated by either proinflammatory or anti-inflammatory stimuli and that ANXA1/FPR2 signalling confers protective effects via inhibiting p38MAPK-associated inflammatory cascade [133]. 

Although clear evidence of a role of MAPKAP kinase 2/3/5 and phosphor-HSP-27 in controlling inflammatory processes is lacking, many studies suggest that HSP-27 can inhibit TNF-α-induced activation of the proinflammatory NF-κB pathway. In fact, phosphorylation of HSP-27 at serine 15, 78, and 82 stimulates its association with IKKβ, but not IKKα, which downregulates NF-κB activation [134]. These observations suggest that phosphorylated HSP-27 acts as a negative regulator of the NF-κB cascade and that MAPKAP kinase 2/3/5-mediated phosphorylation of HSP-27 may stimulate this inhibitory role. In addition, phosphorylation of HSP-27 by MAPKAP kinase 2 allows the interaction between phospho-HSP-27 and p38MAPK. Therefore, the MAPKAP kinase 2-HSP-27 pathway counteracts NF-κB activation through TNF-α-p38MAPK-mitogen- and stress-activated kinase1 signalling [135]. Our results indicate that p38MAPK/HSP-27 pathway is activated by pro-resolving ligands and that it requires NADPH oxidase activity.

**Figure 4:**
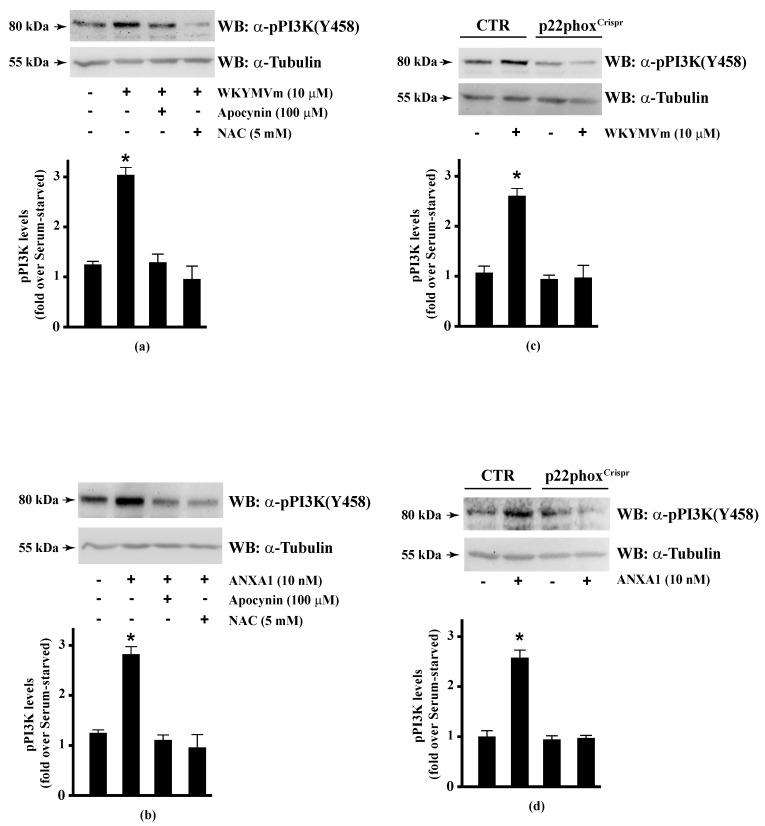
PI3K phosphorylation triggered by WKYMVm or ANXA1 signalling is prevented by NADPH oxidase inhibition. CaLu-6 cells were grown until they reach 80% of confluence, and then serum deprived for 24 h. Cells were stimulated with (**a**) 10 μM WKYMVm or (**b**) 10 nM ANXA1 for 5 min, or preincubated with 100 μM apocynin or 5 mM NAC before stimulation. Serum-starved CaLu-6-control^Crispr/Cas9^ cells (CTR) and p22phox^Crispr/Cas9^ (p22phox^Crispr^) cells were stimulated with (**c**) a scrambled hexapeptide or 10 μM WKYMVm for 5 min, or (**d**) the vehicle or 10 nM ANXA1 for 5 min. Total extracts were electrophoresized on 10% SDS-PAGE and membranes were incubated with an anti-phospho-PI3K (Y458) antibody (α-pPI3K). An antitubulin (α-tubulin) antibody was used as a control for protein loading. Bar graphs show the densitometric analysis performed on phosphorylated bands, as described in Materials and Methods. * *p* < 0.05 as compared with unstimulated cells. The experiments are representative of at least three independent assays.

The serine/threonine-protein kinase OSR1 is activated by WNK protein kinase family, which phosphorylates a Thr185 residue in the T-loop kinase domain and Ser325 and Ser339 residues in the S domain of OSR1. Some evidence suggests that serine phosphorylation plays a functional role in binding to the scaffolding protein MO25 by interacting with OSR1, and thus enhancing its catalytic activities [107]. WNK-OSR1 cascade is activated by PI3K-Akt signalling [136]. PI3K phosphorylates Akt in the activation loop (Thr308) and Akt phosphorylates WNK at Thr60 residue [137]. Interestingly, phospho-OSR1(Ser339) has been identified as a phospho-protein involved in the signalling networks downstream to mTORC1 and mTORC2 [138,139]. mTORC2 phosphorylates both Akt in the C-terminal hydrophobic motif (Ser473) and OSR1 at Ser339 residue [140]. Therefore, the phosphorylation at Ser339 residue of OSR1 observed in WKYMVm- and ANXA1-stimulated CaLu-6 cells suggests that the FPR2 pro-resolving agonists trigger the activation of the FPR2-PI3K-Akt-WNK-OSR1 or FPR2-mTORC2-Akt/OSR1 cascades. However, PI3K activation may occur via growth factor receptors such as EGFR [141]. Consequently, we analysed by Western blot PI3K activation and observed that incubation with WKYMVm or ANXA1 induced PI3K(p85) Tyr458 phosphorylation [142], which was prevented by preincubation with apocynin or NAC (Figure 4, Panels a, b). The incubation of p22phox^Crispr/Cas9^ cells with the two FPR2 agonists did not induce PI3K(p85) phosphorylation (Figure 4, Panels c, d). 

The activation of NADPH oxidase by PI3K/Akt signalling contributes to increase ROS levels in several cell types [143] and is required to promote cell migration and chemotaxis in response to various stimulants [144,145]. Consequently, inhibition of the PI3K/Akt pathway also prevents the translocation of NADPH oxidase subunits and reduces ROS production. Higher levels of ROS affect activation of PI3K/Akt signalling, mainly through inhibition of phosphatases or by direct activation of oncogenes such as Akt [146,147,148]. For instance, ROS can inhibit phosphatidylinositol-3,4,5-trisphosphate 3-phosphatase (PTEN) activity [149], through oxidation of cysteine residues leading to the reversible formation of disulfide bridges and reduction of PTEN catalytic activity [150,151]. ROS can modulate PI3K/Akt signalling also by regulating protein tyrosine phosphatases (PTPase) that inhibit TKRs activity through their dephosphorylation. In fact, ROS have been shown to reversibly oxidize cysteine residues of PTPase, leading to reduced phosphatase activity and sustained TKR activation [152]. In previous works, we demonstrated that FPR stimulation triggers NADPH oxidase-dependent transactivation of several TKRs, such as EGFR, c-Met, VEGFR2, and neurotrophin receptor TrkA, and that the activation of their respective cytosolic phosphorylated tyrosines provide docking sites for recruitment of PI3K/Akt and other signalling proteins [15,16,17,20].

NAC and apocynin have prevented PI3K activation in several experimental systems [153,154,155,156]. NAC increases cellular synthesis of glutathione and can reduce superoxide generation [157]. However, NAC has biphasic effects since ROS generation is increased at low concentrations of NAC, but it is reduced at higher concentrations [158]. Furthermore, apocynin exhibits uncharacterized effects not related to NADPH oxidase inhibition. A pro-oxidant activity of this drug, observed in mouse embryonic stem cells, suggests a possible unexpected interaction of this NADPH oxidase inhibitor with intracellular signalling pathways [159]. The results we obtained in p22phox^Crispr/Cas9^ cells show that PI3K phosphorylation is completely prevented upon pro-resolving FPR2 agonists stimulation, thus, suggesting that it depends on NADPH oxidase activation.

**Figure 5:**
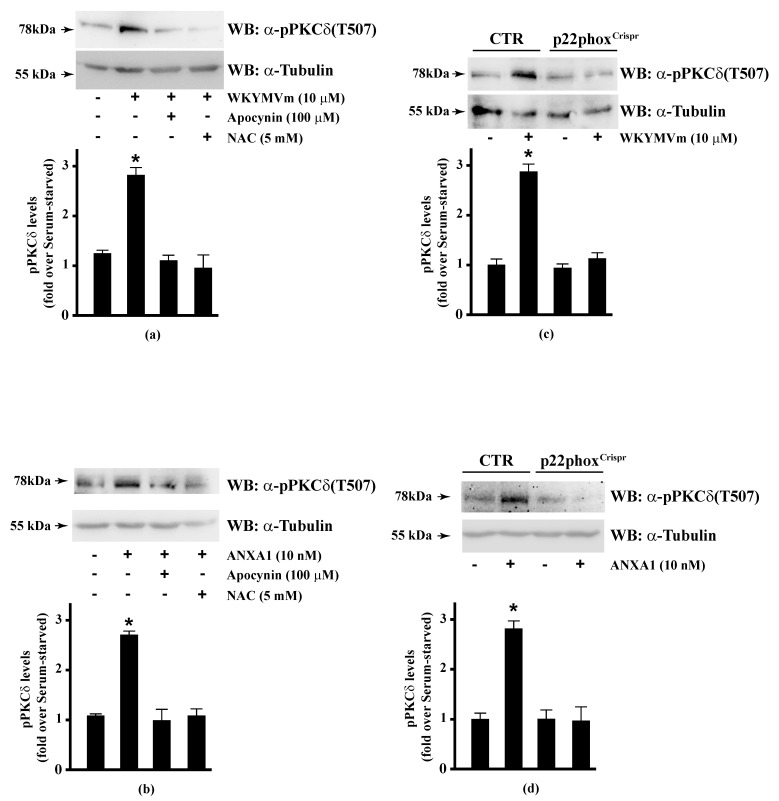
WKYMVm- and ANXA1-induced PKCδ activation depends on NADPH oxidase-dependent ROS generation. Serum-starved CaLu-6 cells were stimulated with (**a**) 10 μM WKYMVm or (**b**) 10 nM ANXA1 for 5 min, in the presence or absence of the appropriate amounts of NADPH oxidase inhibitors. CaLu-6-control^Crispr/Cas9^ cells (CTR) and p22phox^Crispr/Cas9^ (p22phox^Crispr^) cells were serum deprived for 24 h, and then stimulated with (**c**) a scrambled hexapeptide or 10 μM WKYMVm, or (**d**) the vehicle or 10 nM ANXA1 for 5 min. Sixty micrograms of whole extracts were resolved on 10% SDS-PAGE, transferred onto PVDF membrane, and incubated with an anti-phospho-PKCδ(Thr507) antibody ((α-pPKCδ(T507)). An antitubulin (α-tubulin) antibody was used as a control for protein loading. Bar graphs show the densitometric analysis performed on phosphorylated bands. Data are representative of at least three independent experiments. * *p* < 0.05 as compared with unstimulated cells.

PKCα, PKCδ, and PKCε phosphorylate Ser159, Ser163, and Ser170 residues in the phosphorylation site domain of MARCKS. The negative charges of the phosphorylated residues mask positive charges on the protein, segregating MARCKS from the plasma membrane to the cytoplasm [160] and, in turn, CaM accessibility [112]. FPR1 and FPR2 stimulation activates PKCα and PKCδ [15,16,49,82,115,161] and rottlerin, a PKCδ inhibitor, prevents FPR1- and FPR2-dependent MARCKS phosphorylation [56,114]. Therefore, we analysed the effects of NADPH oxidase blockade on PKCδ activation in FPR2-stimulated CaLu-6 and p22phox^Crispr/Cas9^ cells. We found that apocynin and NAC prevented WKYMVm- or ANXA1-induced PKCδ phosphorylation at Thr507 residue in the activation loop of the kinase domain, which is required for PKCδ activation (Figure 5, Panels A, B). The same results were obtained in p22phox^Crispr/Cas9^ cells (Figure 5, Panels C, D). 

PKC isoenzymes represent a group of cell-signalling molecules that are sensitive targets for redox modification [162]. They can be activated by oxidative stress and inhibited by antioxidants [163]. In fact, NADPH oxidase inhibition prevents the activation of several PKC isoforms, such as PKCα, PKCβI, PKCβII, PKCγ, and PKCε [164]; moreover, NOX4 deletion results in PKCα and PKCβI inhibition [165]. Furthermore, apocynin inhibits hyperglycaemia-dependent PKCβ, PKCβII activity [166], and N-fMLP-induced PKCδ activation [15].

Both catalytic and regulatory domains of PKC contain cysteine-rich regions, which make this family of isoenzymes a target for oxidation-reduction regulation. Reversible oxidative modifications of cysteine residues in PKC isoenzymes alter their kinase activity, suggesting thiol-directed redox mechanisms in PKC regulation [167]. It should be highlighted that PKC isoenzymes are subjects to a dual redox regulation in which oxidants can stimulate or inactivate the kinase activity depending on their concentration. Higher concentration reacts with catalytically cysteine residues and inactivate the enzyme, whereas low oxidant doses stimulate PKC activity [168,169]. Oxidants enhance PKCδ activity by phosphorylation at Tyr512 and Tyr523 residues in subdomain VIII [170], which is not a mechanism of activation commonly observed for protein kinases related to the PKC family. Therefore, PKCδ could be phosphorylated on both Thr507 and tyrosine residues upon stimulation with oxidants, suggesting a cooperation between these phospho-sites in amplifying the signal triggered by oxidants. Antioxidants can inactivate PKC by oxidizing vicinal thiols present in the catalytic domain of the kinase that are required for PKC activity, thus, counteracting the effects of oxidants [171].

Overall, our results strongly suggest that some signalling pathways triggered by pro-resolving FPR2 agonists depend on NADPH oxidase activity.

## 4. Conclusions

Our results show that pro-resolving FPR2 agonists WKYMVm and ANXA1 induce HSP-27(Ser82), OSR1(Ser339), and MARCKS(Ser170) phosphorylation, as well as the activation of p38MAPK, PI3K and PKCδ, the kinases upstream to HSP-27, OSR1 and MARCKS, respectively. NADPH-derived ROS play a key role in the signalling cascade triggered by the two pro-resolving FPR2 agonists. In fact, we demonstrate that blockade of NADPH oxidase functions prevents HSP-27, OSR1, and MARCKS phosphorylation and the activation of the upstream kinases.

NADPH oxidase-derived ROS have emerged as key regulators of inflammatory pathways. High levels of ROS have been associated with oxidative stress in multiple chronic inflammatory disorders [172], even though it is unclear whether oxidative stress is a cause or consequence of inflammation associated with these disorders. In addition, patients affected by chronic granulomatous disease (CGD), which have deficiency in NADPH oxidase activity, are highly susceptible to inflammatory complications. In agreement, mice lacking NADPH oxidase activity show hyper-inflammatory responses characterized by elevated levels of proinflammatory cytokines [173]. 

FPR2 is expressed in several cells and tissues and its stimulation triggers NADPH oxidase activation, allowing the timely generation of ROS. FPR2 is the most versatile member of the FPR family and can interact with a multitude of ligands resulting in both anti-inflammatory (ANXA1), pro-resolving (LXA4), and proinflammatory (serum amyloid A, SAA, and cathelicidin) functions. The switch between proinflammatory and anti-inflammatory responses is caused by conformational changes adopted by FPR2 after its association with a specific agonist and by the ability of FPR2 to homodimerize or to heterodimerize with the other members of the FPR family. FPR2-specific biased agonists selectively trigger one signalling cascade and induce a restricted functional response. Characterization of its pharmacological properties strongly suggests a prominent anti-inflammatory role for FPR2, and our results show that pro-resolving ligands WKYMVm and ANXA1 can activate the receptor by transducing specific downstream signals and phosphorylating specific kinases through NADPH oxidase-dependent ROS generation. 

Phosphorylation is the result of the balanced action of protein kinases and protein phosphatases and any deviation from their balance, as well as any impairment of their activation, represents one of the most common causes of several human diseases. Protein kinases mediate the interconnection of highly complex signals. Therefore, the comprehension of the regulatory functions of protein kinases may represents a useful tool to identify more successful drugs against human diseases. 

Chronic inflammation is a component of several diseases including autoimmune, metabolic, neurodegenerative, cardiovascular, and cancer. The discovery and characterization of pro-resolving mediators critical to the resolution of inflammation, and their cognate GPCRs has led to a significant increase in the understanding of this pathological process and for developing a new arm of pharmacology. Therapeutic efforts in targeting these receptors are challenging, with very few ligands progressing through to preclinical or clinical development. To date, some knowledge gaps remain in the understanding of how the activation of these receptors, and their downstream signalling, results in efficient resolution of the inflammation. Therefore, characterization of FPR2 pharmacological properties and of NADPH oxidase-dependent intracellular signalling triggered by this receptor may contribute to develop novel target for inflammation therapeutic intervention.

## Data Availability

The data presented in this study are available within the article and its supplementary material. Other data that support the findings of this study are available upon request from the corresponding authors.

## Supplementary Materials

The following are available online at https://www.mdpi.com/2076-3921/10/1/134/s1, Figure S1 p22phox expression.
