# Supplementary material for: Pro-Resolving FPR2 Agonists Regulate NADPH Oxidase-Dependent Phosphorylation of HSP27, OSR1, and MARCKS and Activation of the Respective Upstream Kinases
Source: Antioxidants (Basel). 2021 Jan 19;10(1):134. doi: 10.3390/antiox10010134 (PMC7835750; doi:10.3390/antiox10010134)

Figure 1A

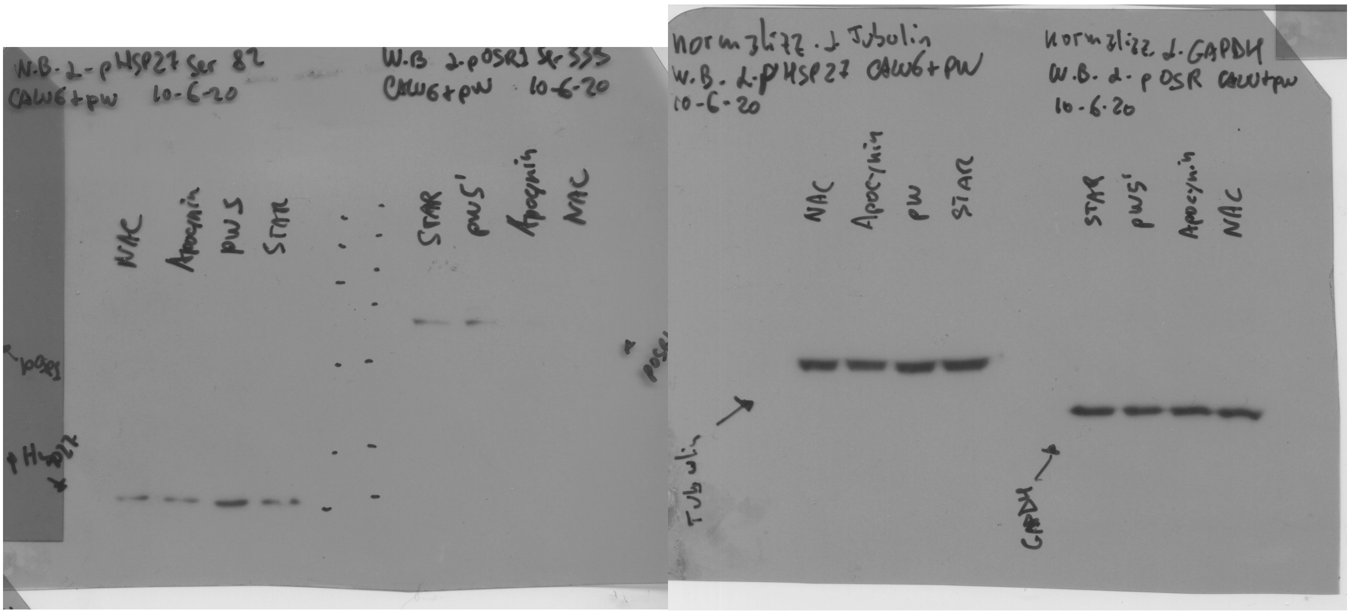

Figure 1b

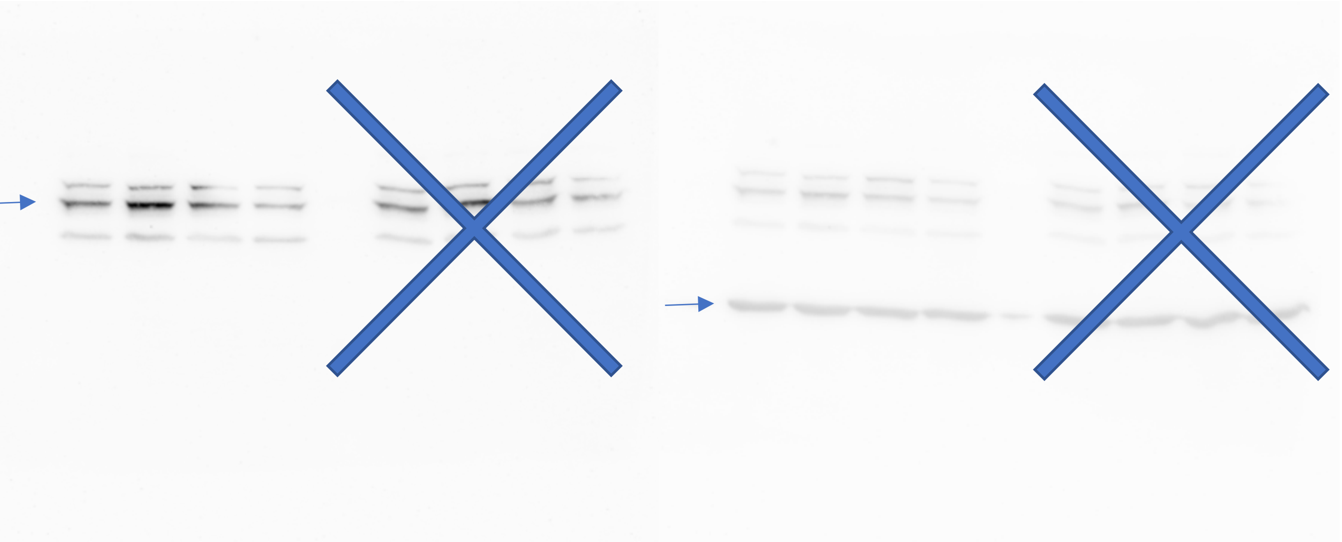

Figure 1c

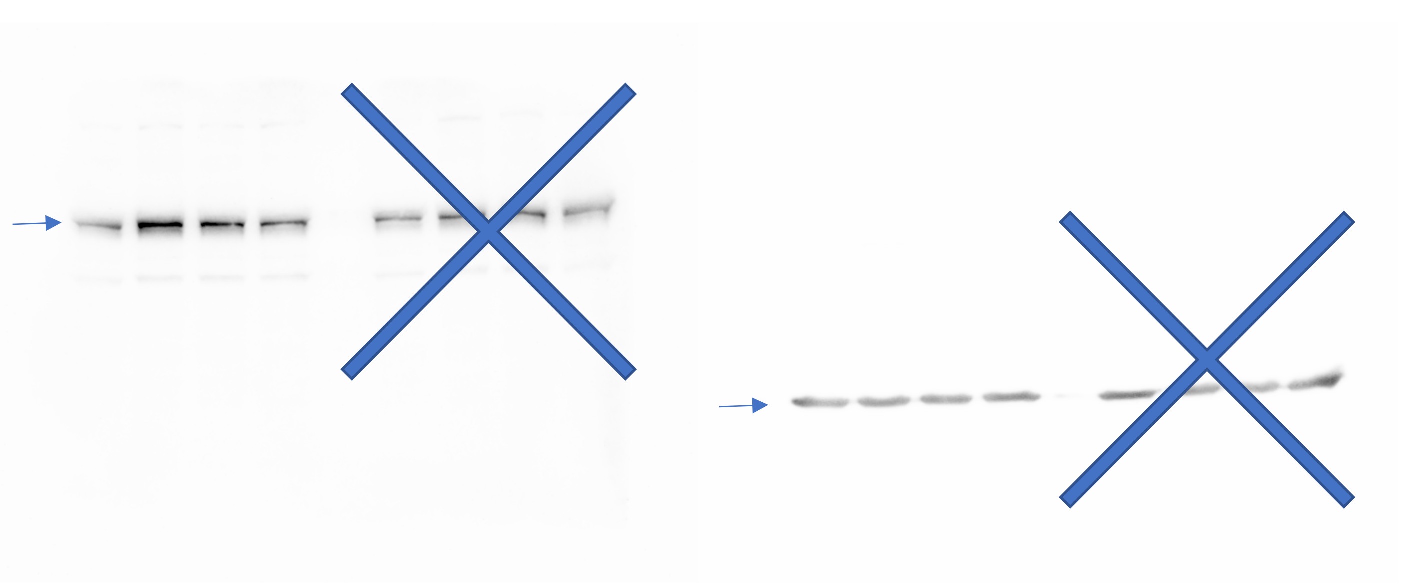

Figure 1d

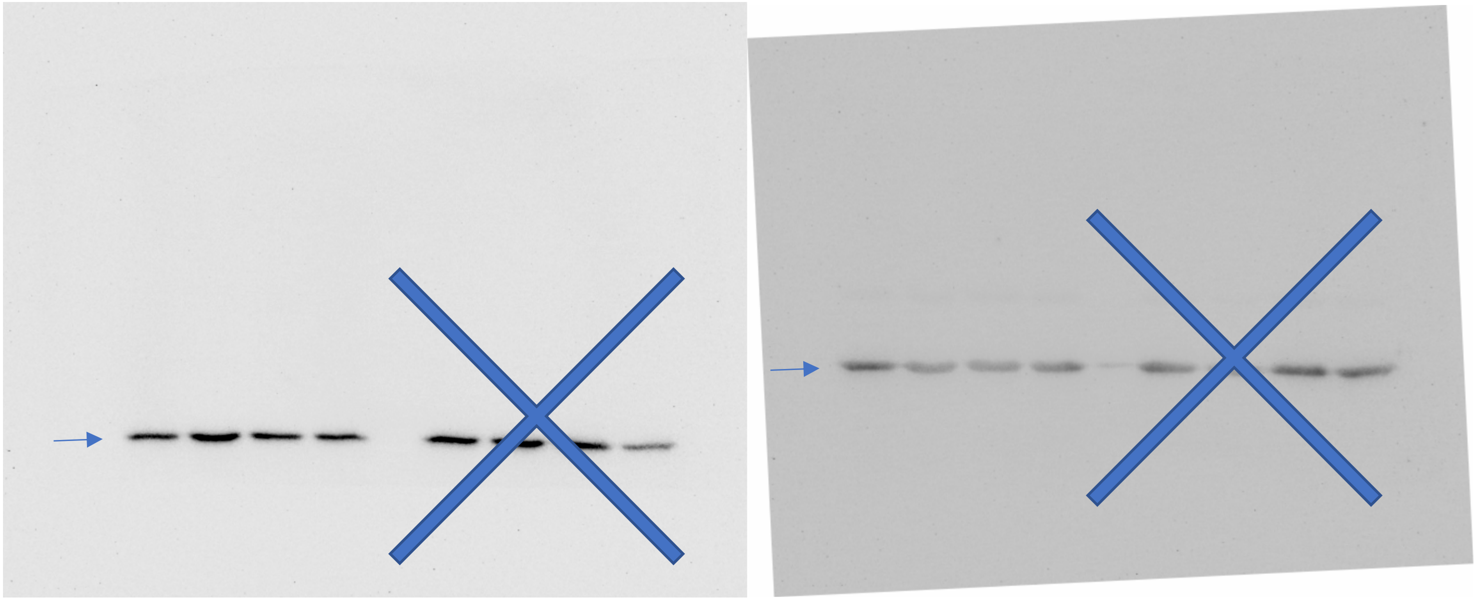

Figure 1e

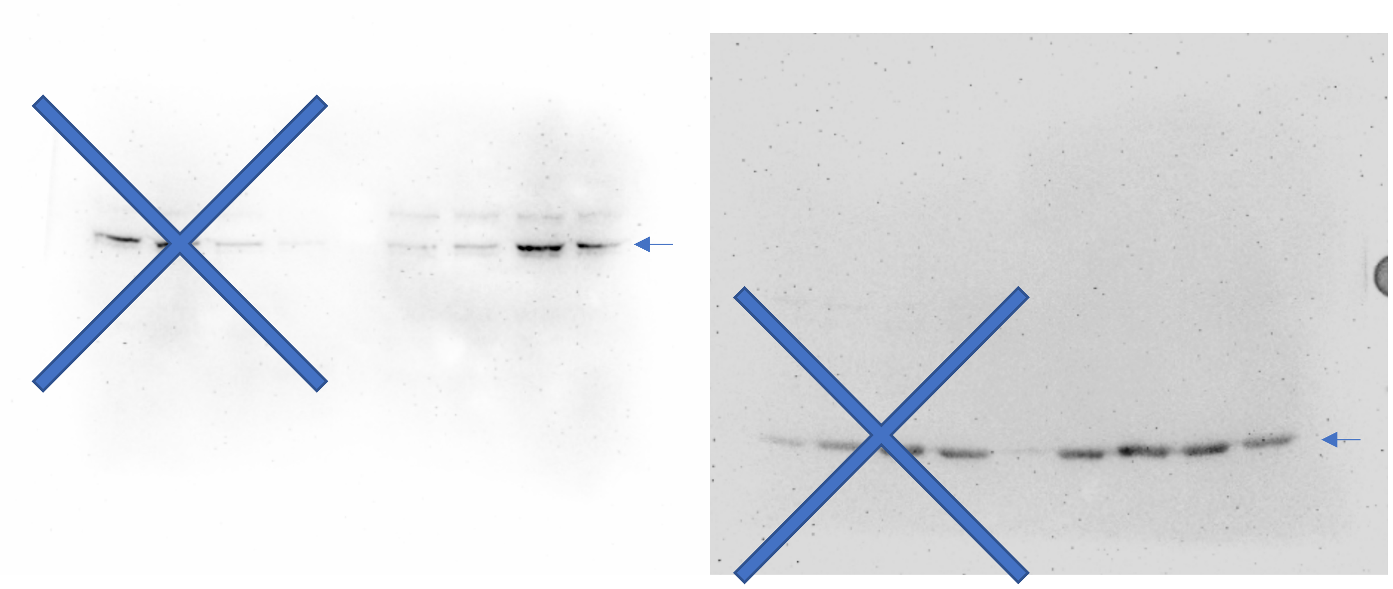

Figure 1f

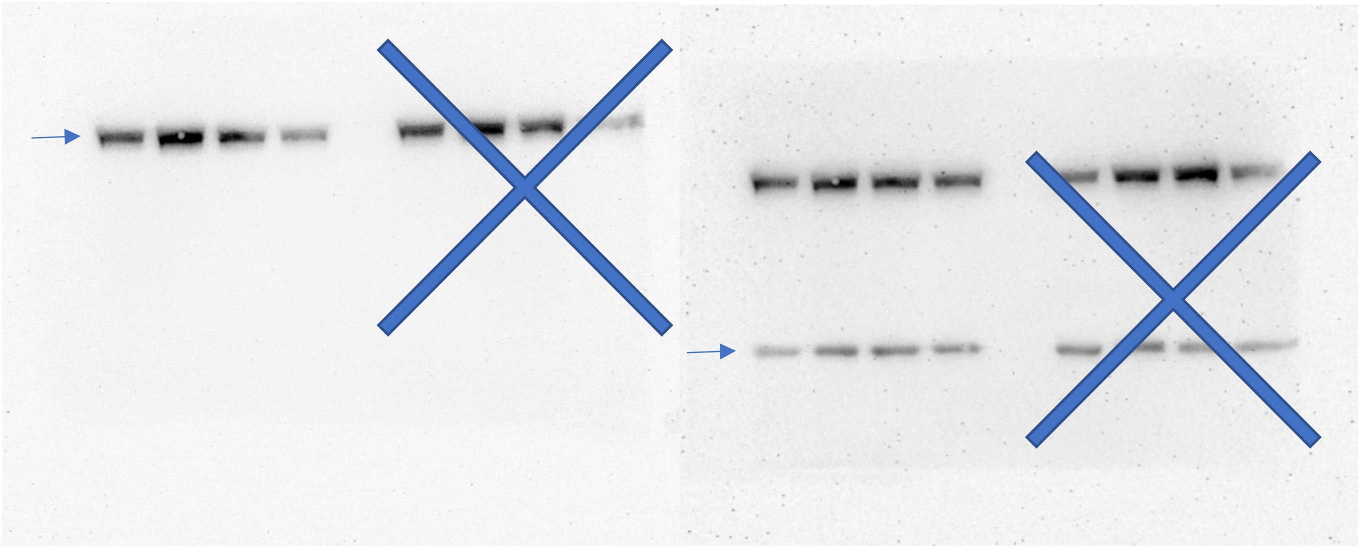

Figure 2a

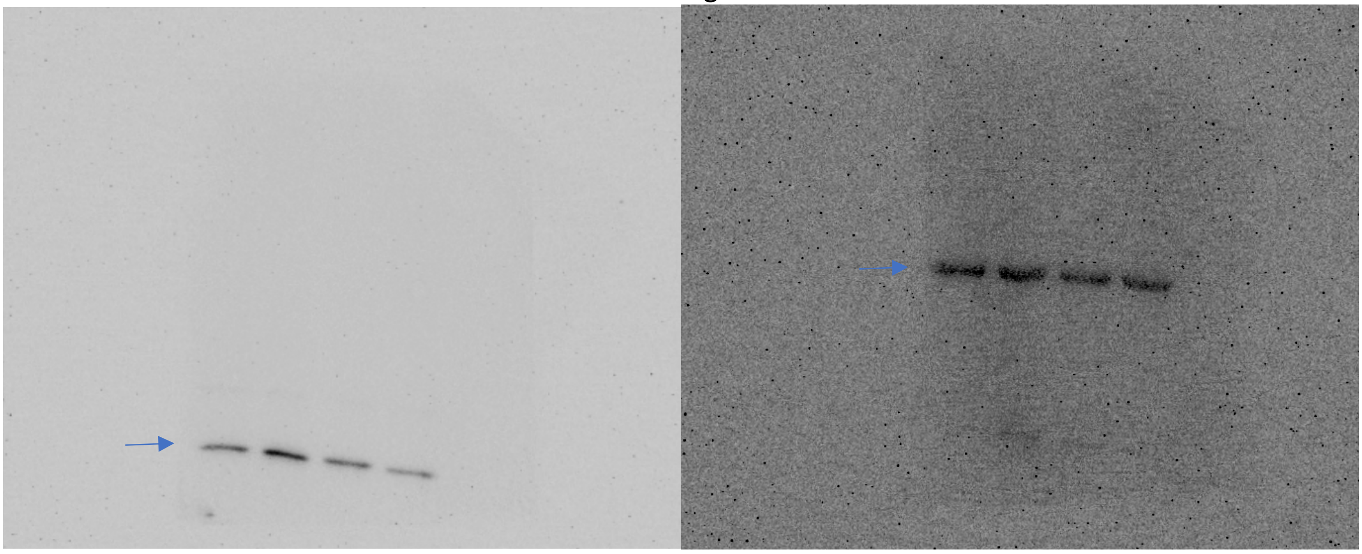

Figure 2b

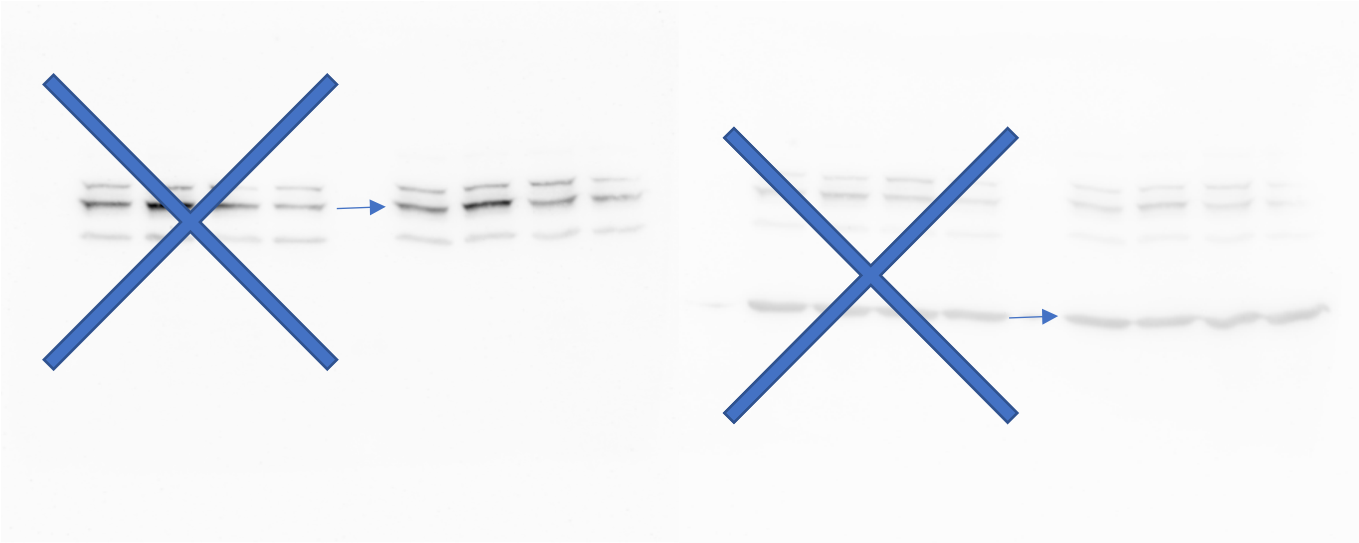

Figure 2c

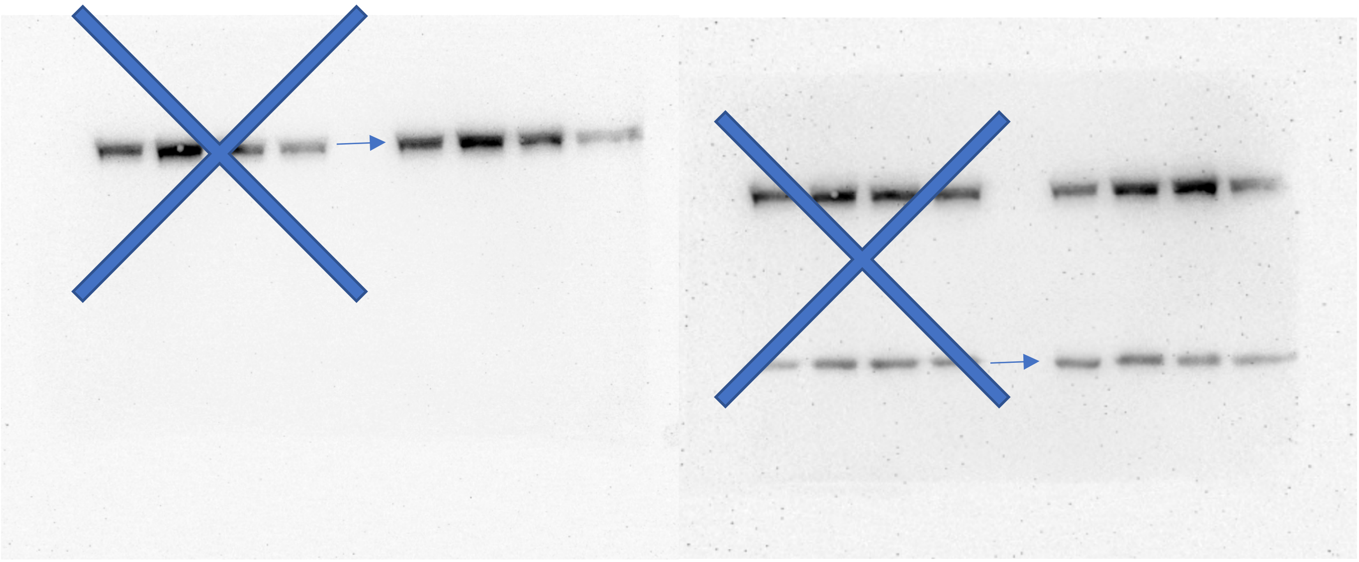

Figure 2d

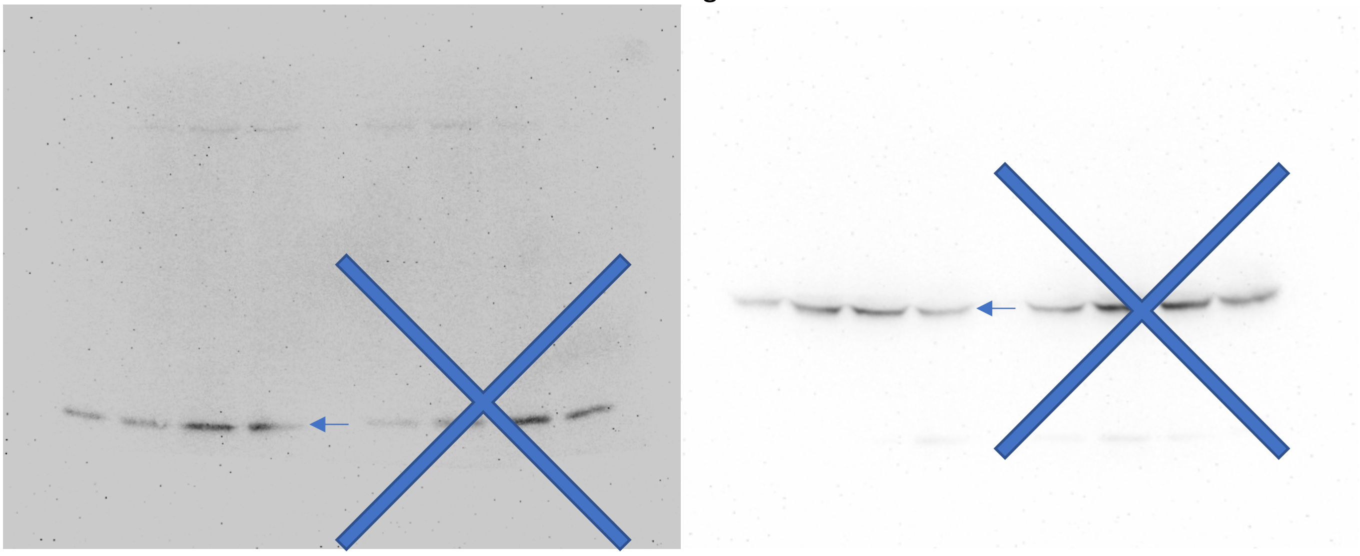

Figure 2e

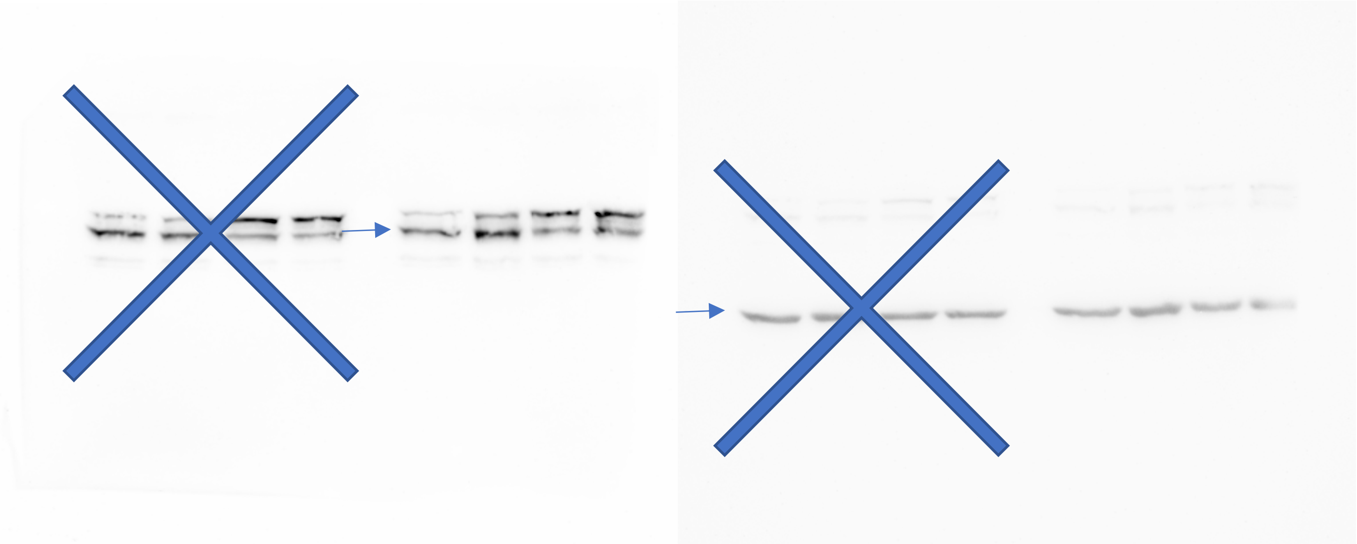

Figure 2f

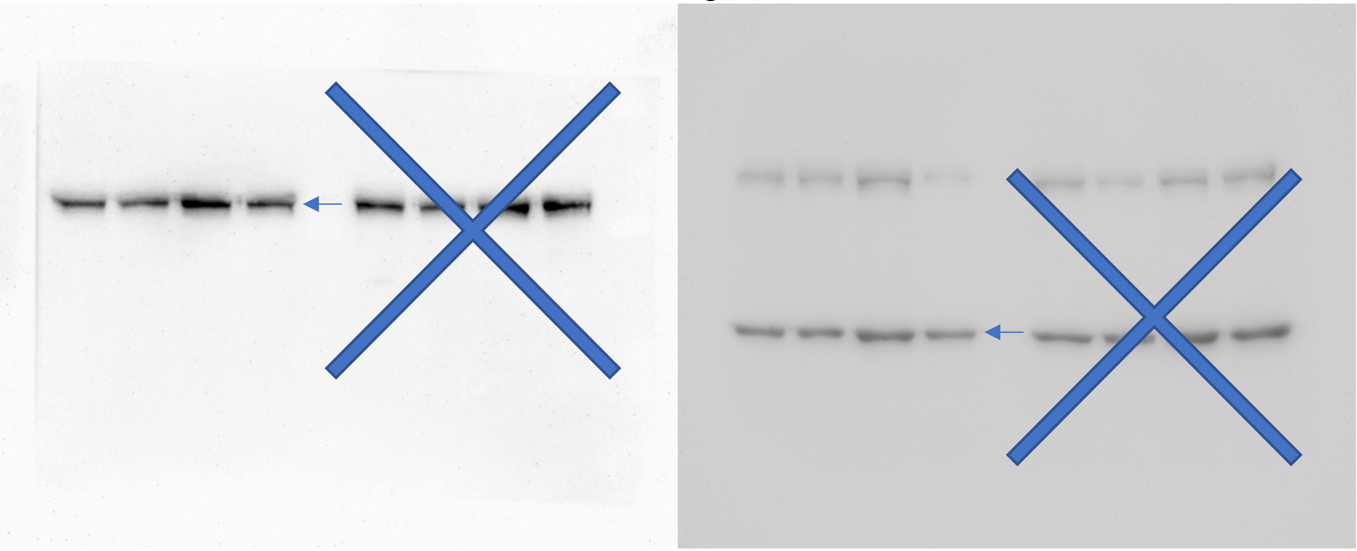

Figure 3a

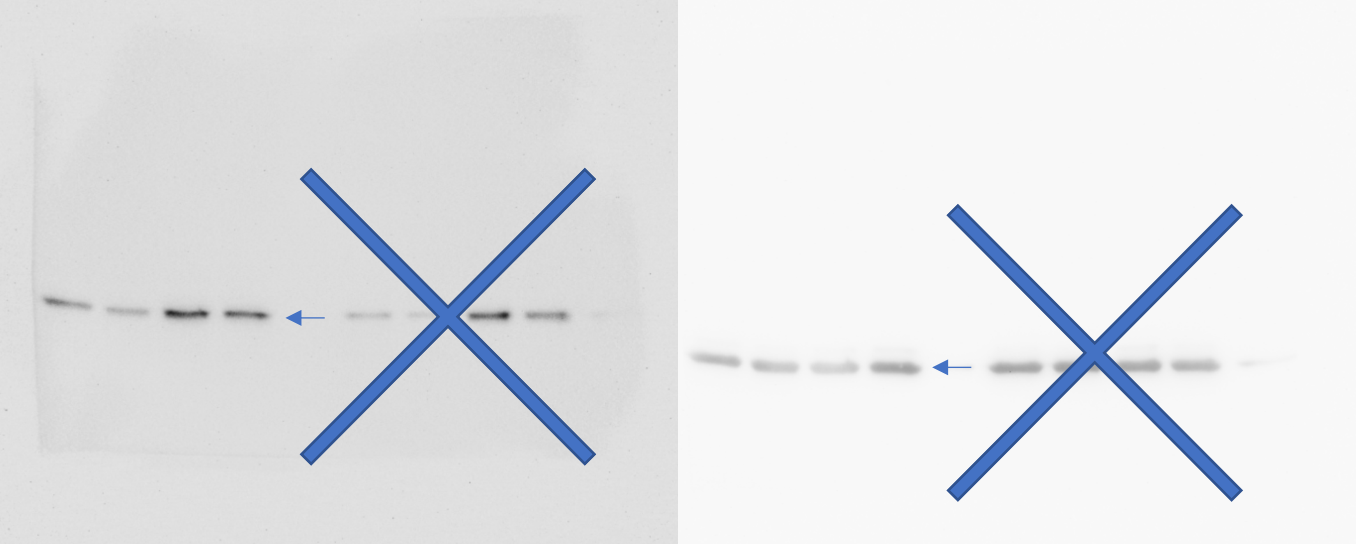

Figure 3b

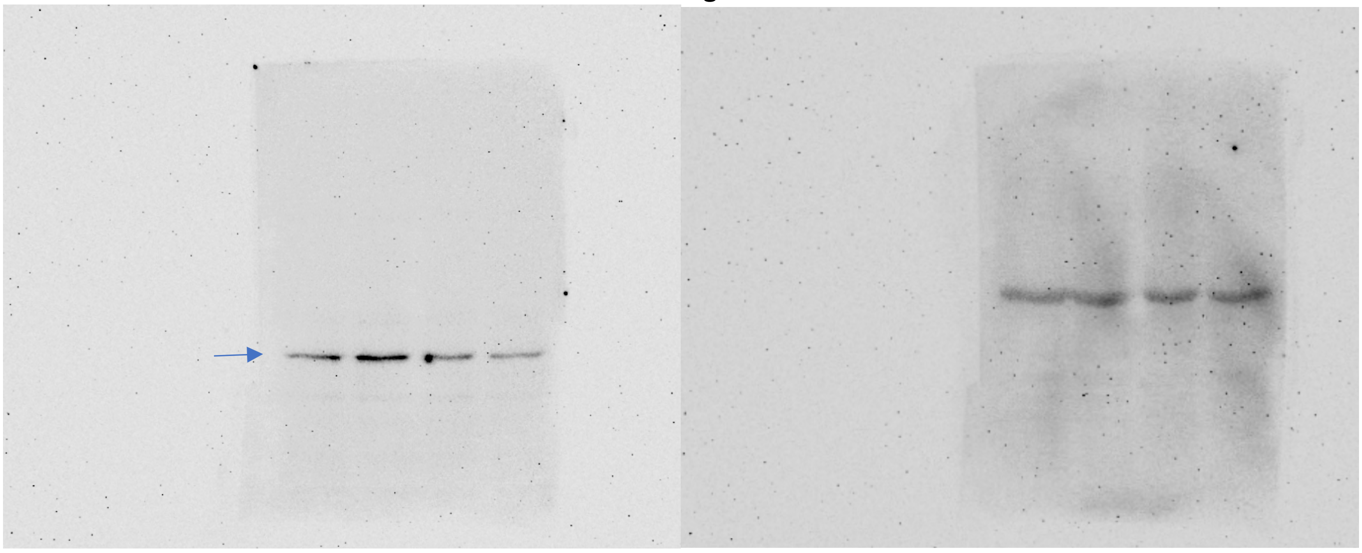

Figure 3c

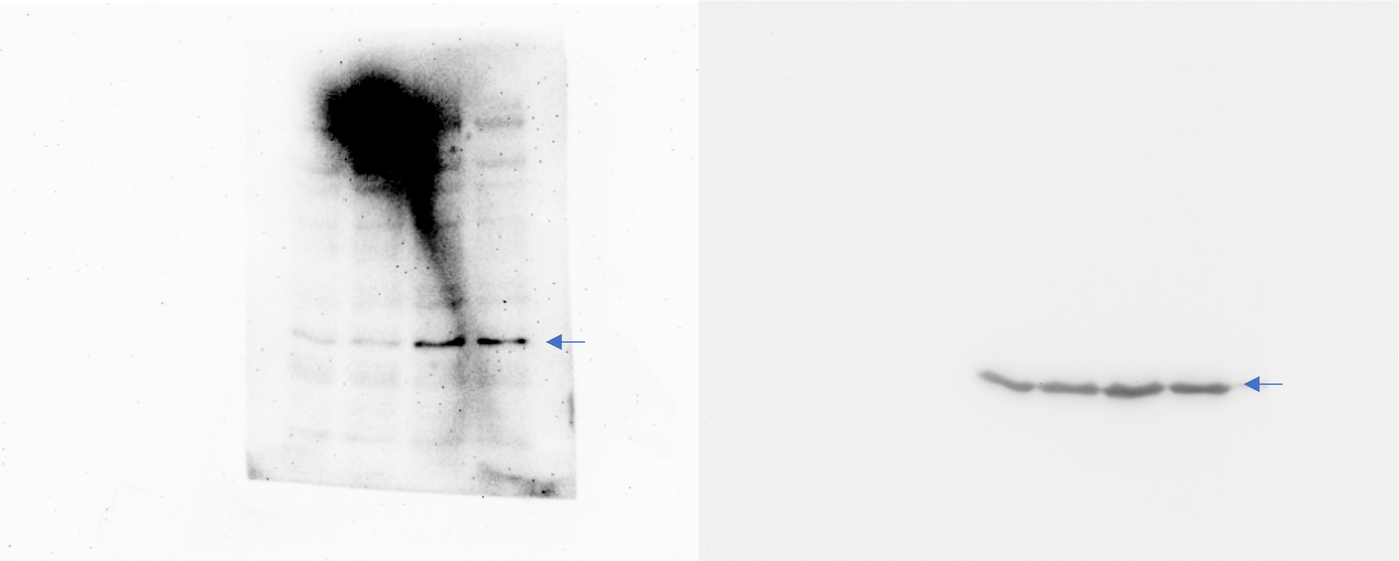

Figure 3d

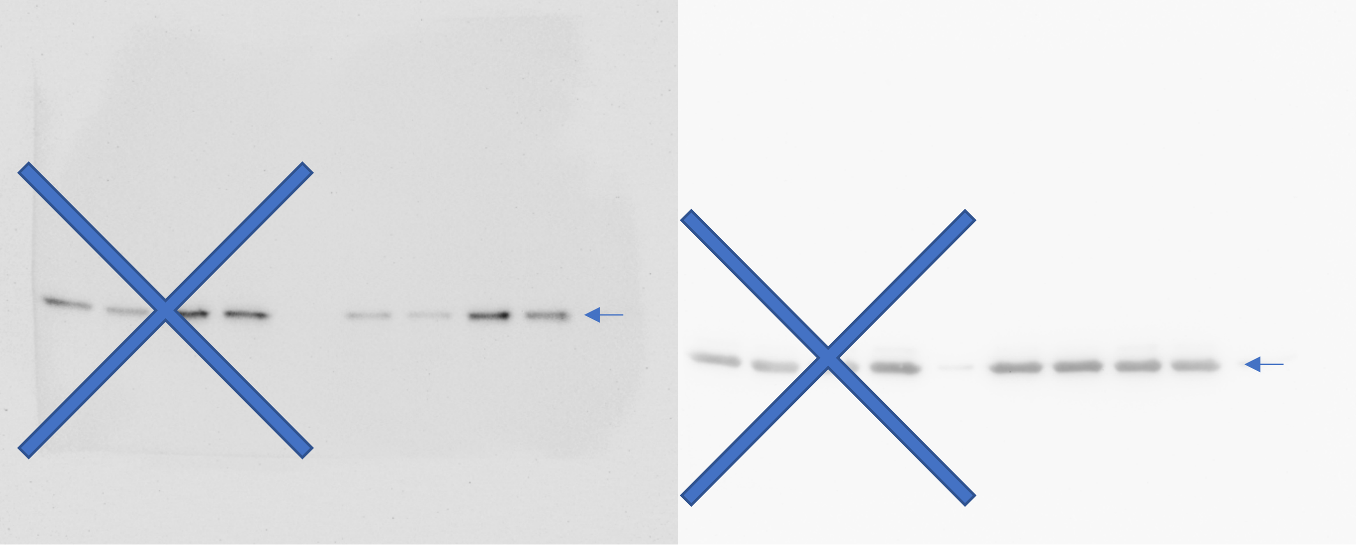

Figure 4a

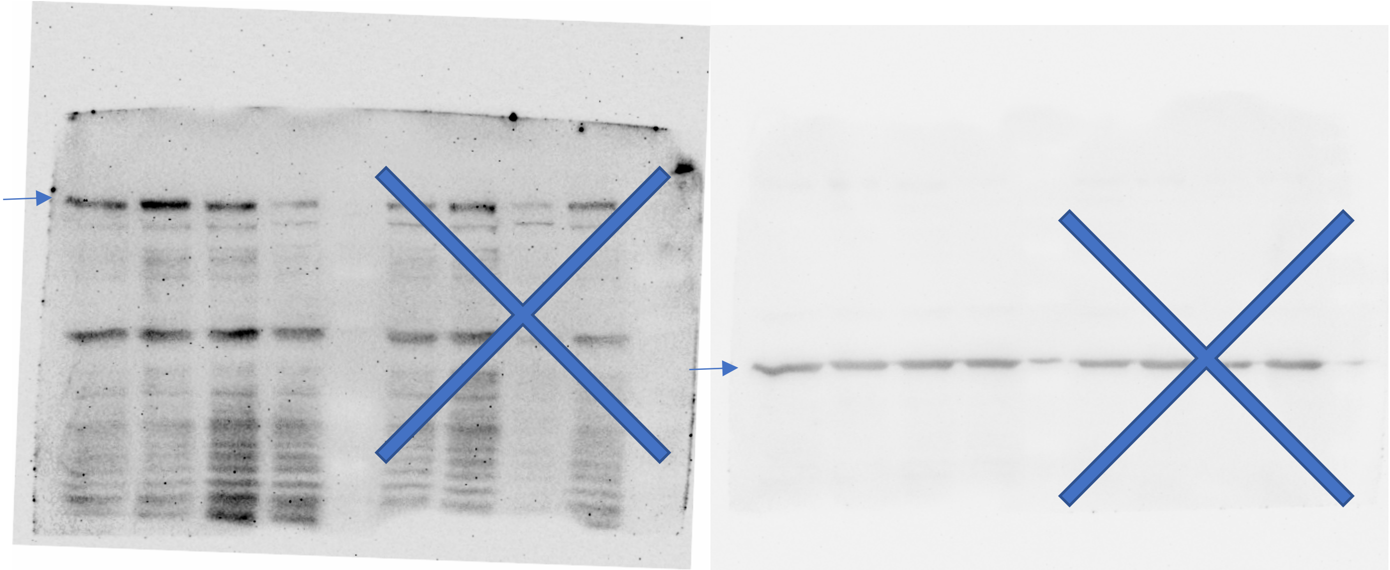

Figure 4b

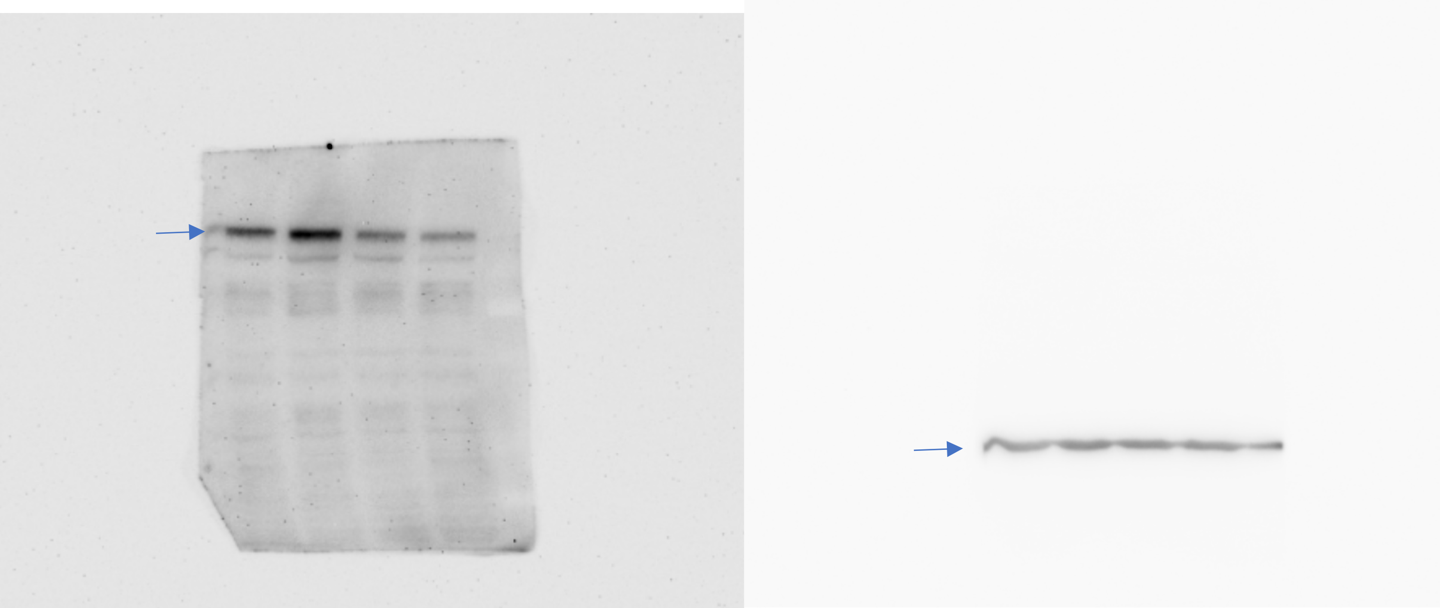

Figure 4c

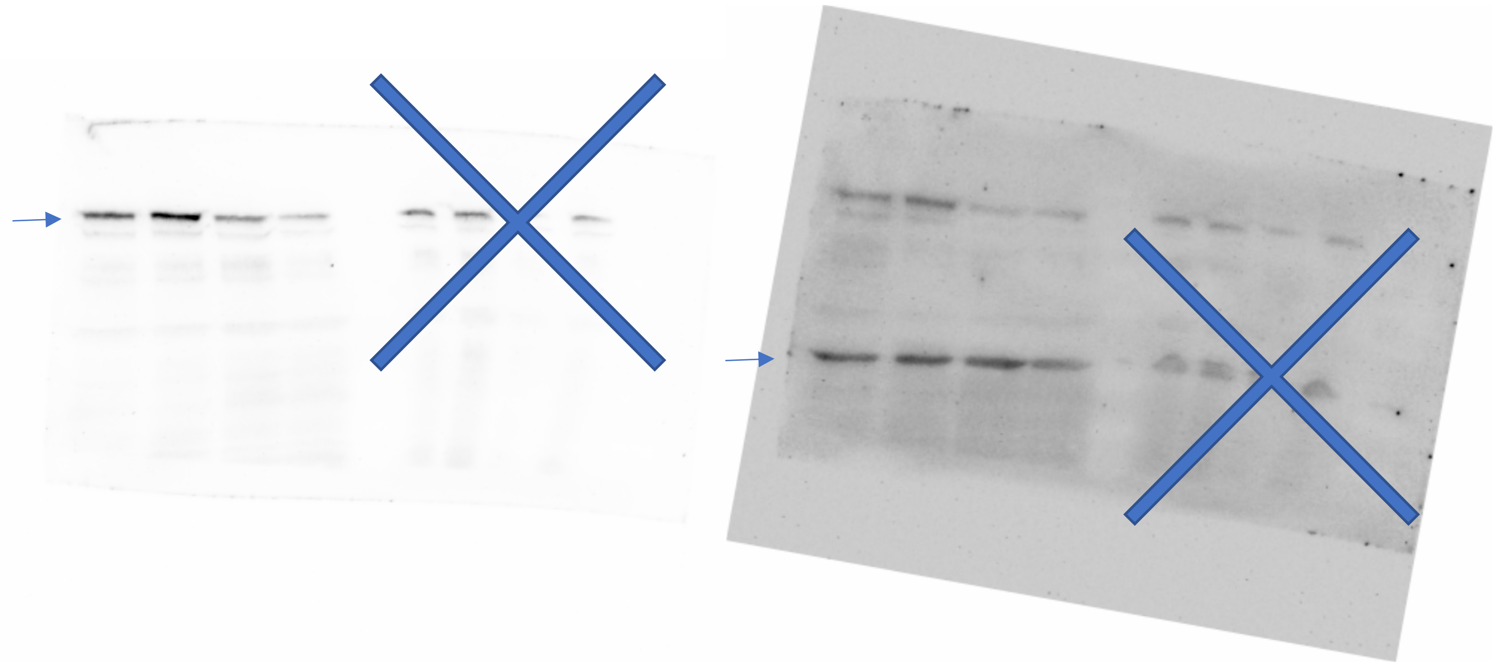

Figure 4d

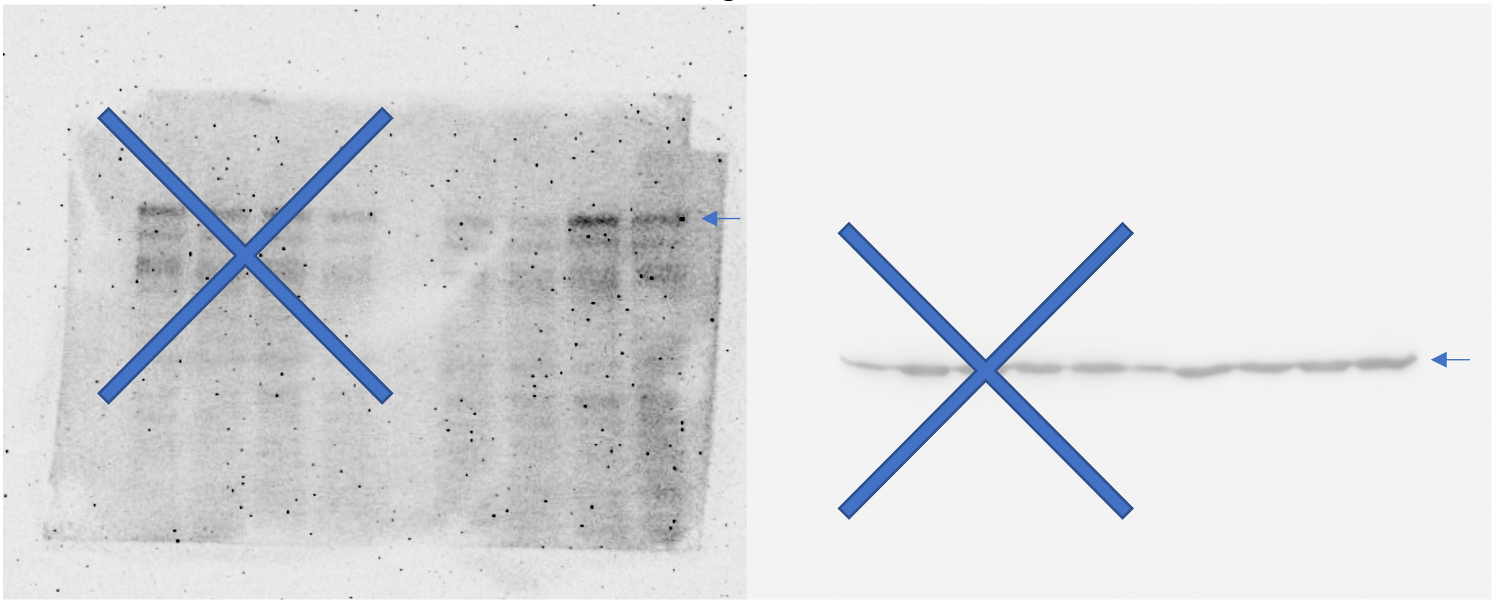

Figure 5a

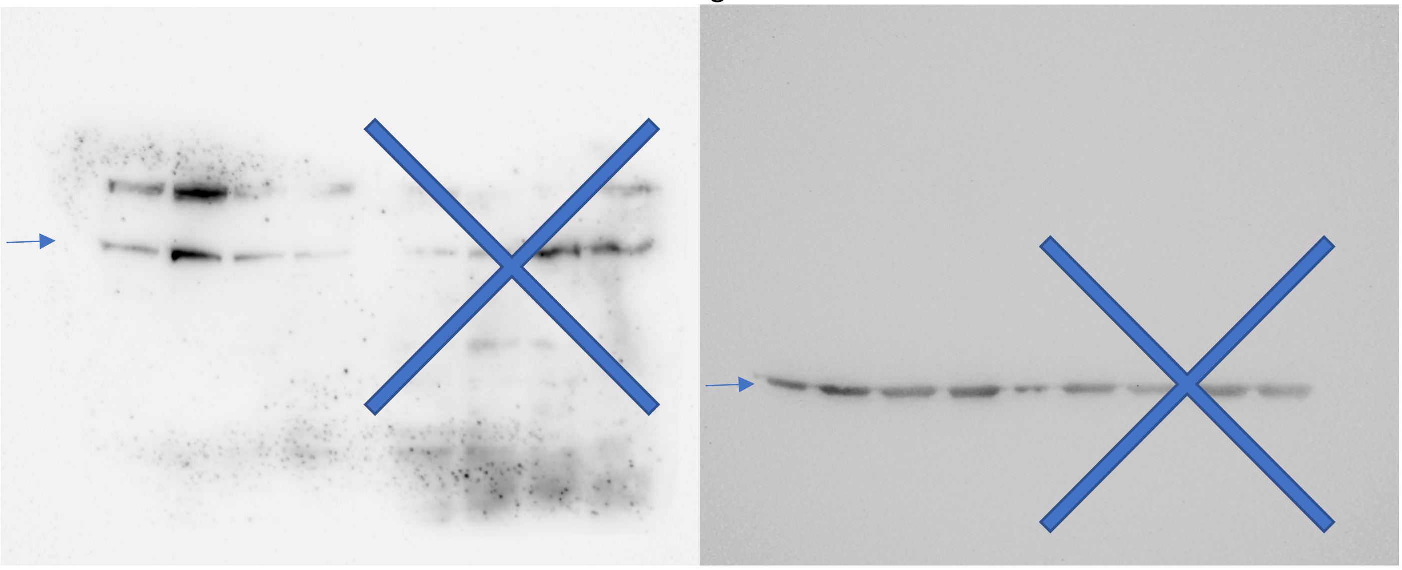

Figure 5b

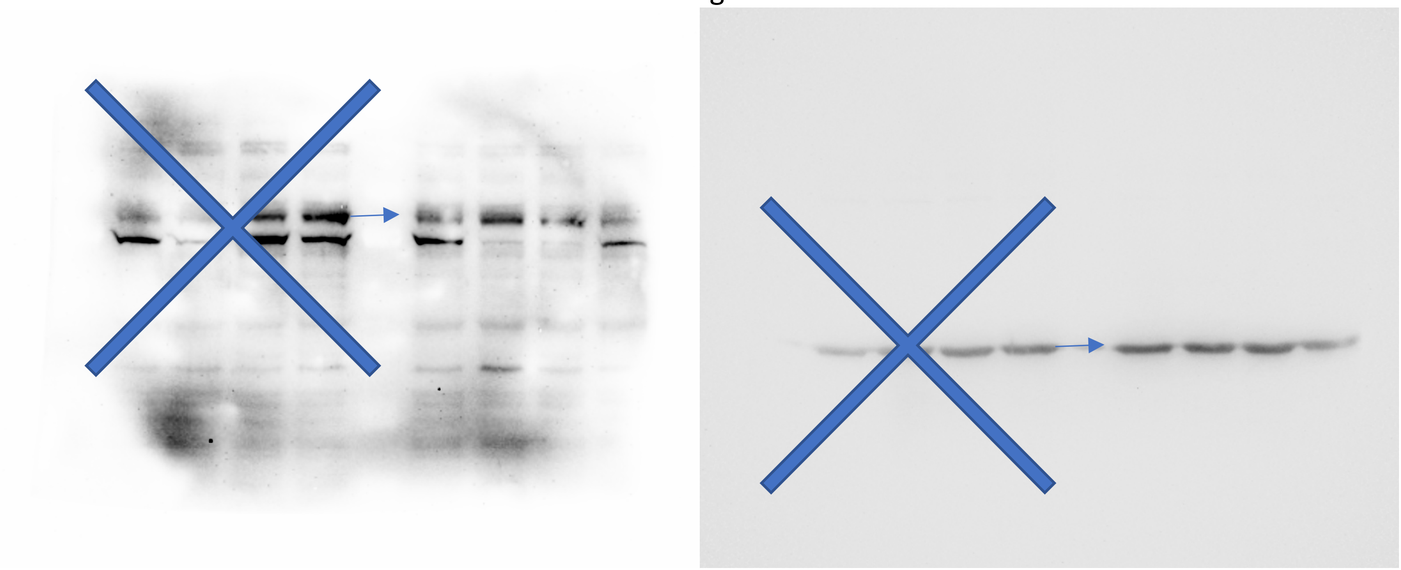

Figure 5c

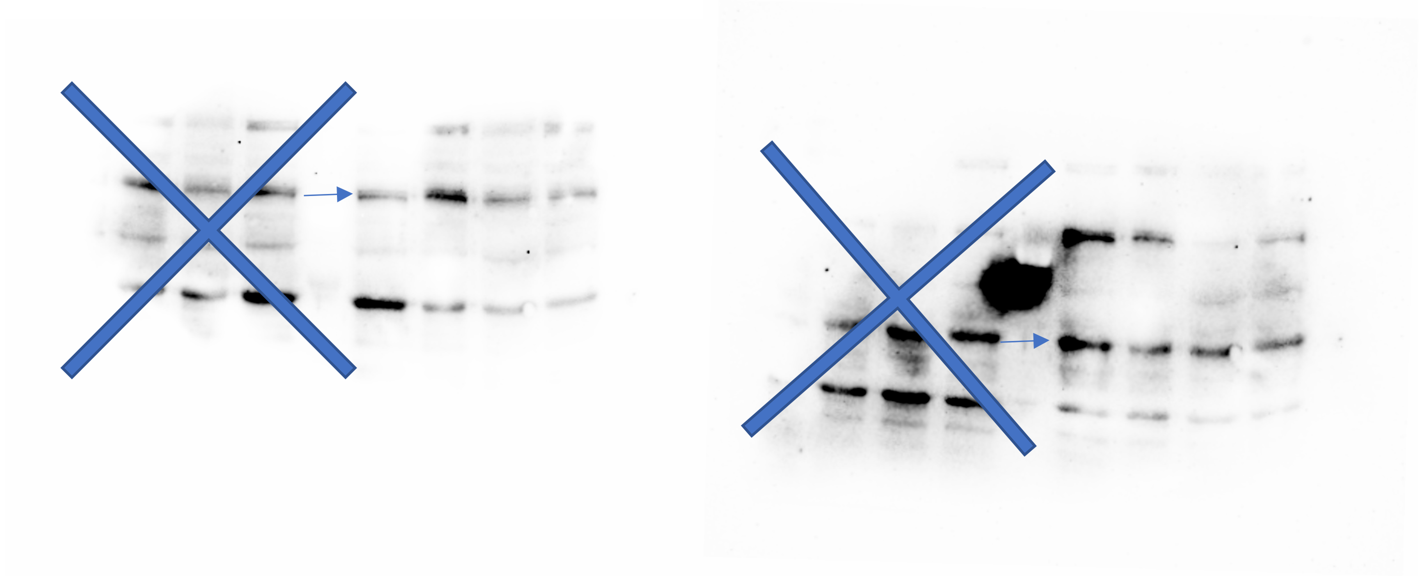

Figure 5d

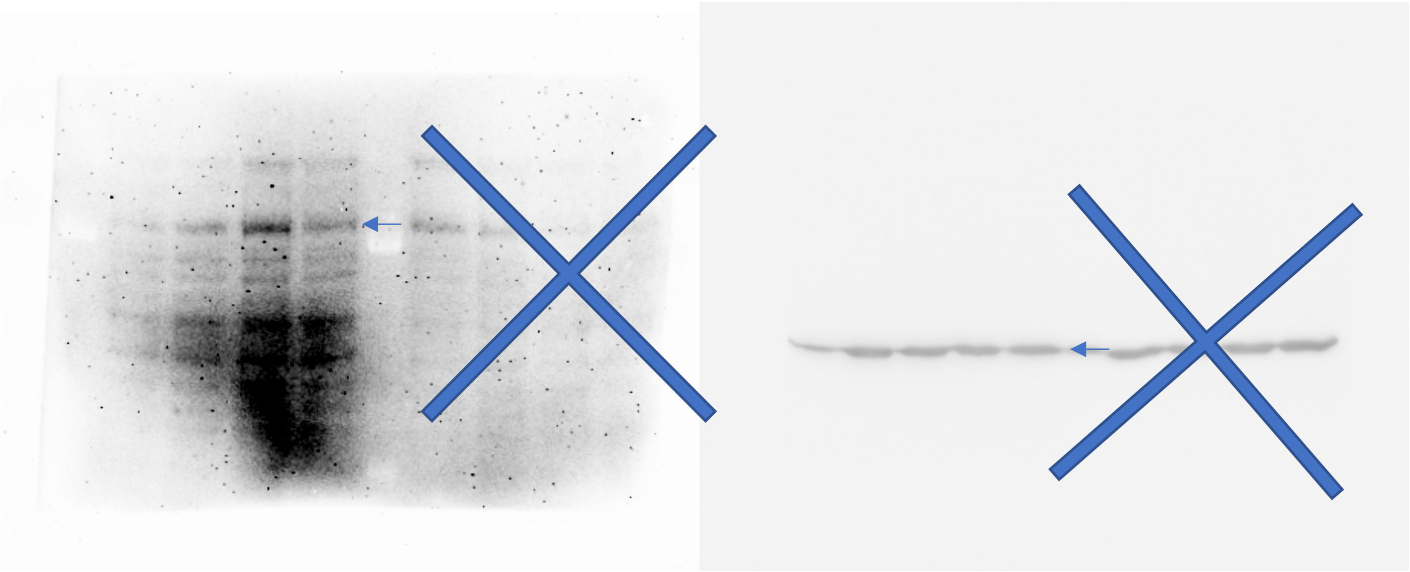

FigureS1

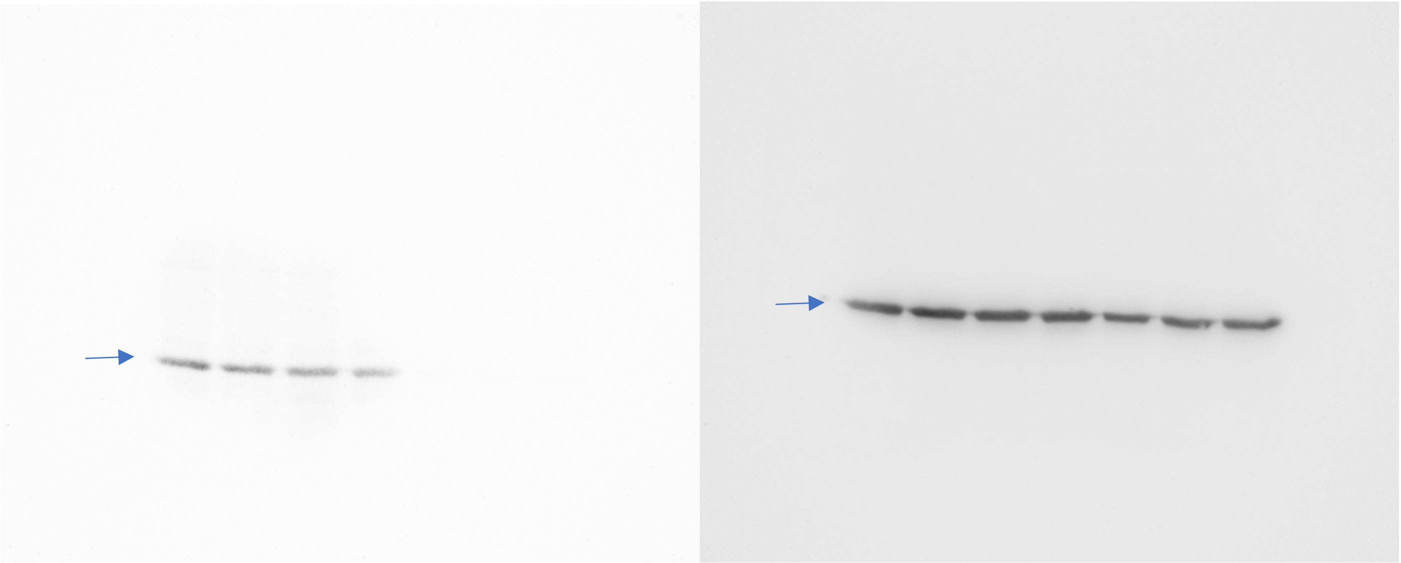

Supplement: Supplementary file 1 [file antioxidants-10-00134-s001.pdf]
